# Unlocking Nature’s Potential: Ferritin as a Universal Nanocarrier for Amplified Cancer Therapy Testing via 3D Microtissues

**DOI:** 10.1021/acsami.4c12524

**Published:** 2024-12-11

**Authors:** Iqra Munir, Faiqa Nazir, Gurkan Yesiloz

**Affiliations:** † National Nanotechnology Research Center (UNAM) Bilkent University, Cankaya, Ankara, 06800, Türkiye; ‡ Institute of Material Science and Nanotechnology, Bilkent University, Cankaya, Ankara, 06800, Türkiye

**Keywords:** nanocarrier materials, protein, drug nanoconjugate, ferritin, 3D microtissues, spheroids

## Abstract

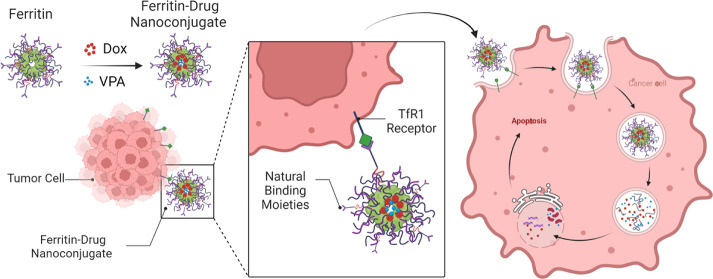

In the existing development of extensive drug screening models, 3D cell cultures outshine conventional 2D monolayer cells by closely imitating the in vivo tumor microenvironment. This makes 3D culture a more physiologically relevant and convenient system in the regime of preclinical drug testing. In the nanomedicinal world, nanoconjugates as nanocarriers are largely hunted due to their capability of precisely binding to target cells and distributing essential dosages of therapeutic drugs with enhanced safety profiles. Thus, for boosted drug availability, the evolution from conventional drug treatment to combination therapies and last switching to drug carriers has gained significant progression in cancer cure. In contrast to conventional engineered nanoparticles, herein, we successfully designed biomolecule (ferritin)-based drug nanoconjugates effective both as a single drug (valproic acid-VPA) and twin-drug (valproic acid/doxorubicin-Dox) carriers, which dramatically enhance the proficiency of the tumor therapeutic modality. To question the reported adjuvant drug property of VPA, we progressed utilizing at first VPA alone as an effective yet exclusive tumor therapy when delivered via some carrier molecule, in particular protein. Subsequently, we paralleled this comprehensive investigation output to compare and test the coloading strategy of drugs and observe the synergistic and/or additive behavior of VPA in conjugation with other anticancer agents (Dox) while given via a carrier molecule. To approach this, VPA and/or Dox molecules were encapsulated into the ferritin (F) cavity using a thermosensitive synthesis method by maintaining the temperature at 60 °C. The successful encapsulation of drugs in the protein nanocage was confirmed through various characterization techniques. The F-VPA/F-VPA-Dox nanoconjugates exhibited similar morphology and structural characteristics to the hollow ferritin cage and showed significant cytotoxicity than the naked drugs when tested on physiologically relevant 3D spheroid models. Precisely, our first designed carrier nanoconjugate, i.e., F-VPA, offered more than a 3-fold increased intratumoral drug concentration than free VPA and significantly suppressed tumor growth after a single-dose treatment. However, our second modeled carrier nanoconjugate, viz. F-VPA-Dox, revealed an extended median survival period and lesser toxicity when administered at a much more effective dose (∼3–5 μM), in 3D tumor spheroid models of various cancer cell lines. All in all, importantly, ferritin nanoconjugates exhibited an enhanced tumor inhibition rate with a single-dose treatment, which further confirms the benefits of the active targeting property of these nanocarriers. Moreover, these nanocarriers also offer to deliver a significant dose of the therapeutic drug into tumor cells, alongside tremendous biocompatibility and safety profiles in numerous tumor 3D spheroid models.

## Introduction

In the prevailing cancer research, 3D cell spheroid models have gained significance over 2D monolayer cells due to their close translation of in vivo microenvironment, including crucial features like gradient constituents, cell–matrix, and cell–cell interactions,^1^ thus making them more physiologically relevant yet convenient in vitro models in the regime of preclinical drug testing. These advancements have also sparked growing interest in the 3D cell culture system as a noticeable tool across various biological disciplines, particularly in cancer research and comprehensive drug screening. While designing 3D culture models, both adherent and nonadherent substrates have been underlined, for instance, via scaffold-based methods in which cells can interact with a substrate or via nonadherent scaffold-free methods in which nonadherent substrates are used to culture cells.^2^ Within the realm of these models, organoids and spheroids have gained significant acceptance, where the former represents self-organized models of complex organ-like tissue,^3^ while the latter represents cell aggregates that form a cell sphere without adhering to any culture substrate. However, in order to attain highly efficient and controllable 3D spheroids, there is a dire need to generate a 3D model system out of various cancer cell lines that offer a state-of-the-art growth pattern monitor for each cell line as a benchmark setup in a controlled and precise manner.

Despite the fact that targeted therapy was first proposed almost a century ago, to date, only a limited number of biomolecule-based nanocarriers and strategies targeting the tumor have progressed to clinical trials. Notably, a few of these carriers have received clinical approval, for instance, Abraxane (albumin-bound paclitaxel), owing to the complexity of synthesizing particles that can meet the intricate criteria for targeted drug delivery. Understanding the current need, herein, we designed a protein–drug nanoconjugate utilizing nature’s multifunctional universal carrier, i.e., ferritin (F), as a drug delivery vehicle. Structurally, this protein is composed of 24 identical subunits, measuring 12 nm externally and forming a robust cage-shaped molecule, ideal to encapsulate different drugs.^4^ Ferritin, a mostly occurring biopolymer in living organisms, raises fewer concerns in clinical translation compared to synthetic nanocarriers. Being a naturally abundant protein involved in iron homeostasis and storage, it offers several advantages for biomedical use. Ferritin-based nanosystems possess favorable properties compared to other systems, including high solubility and stability in water and blood as well as low toxicity. These characteristics make ferritin an appealing natural universal choice for in vivo applications in humans. Besides structure, ferritin also offers a wide range of attractive characteristics like uniform nanosize, easy functionalization, and low cost for large-scale production.^5,6^ Therefore, we aimed to design ferritin–drug nanoconjugates as a single and/or dual drug carrier that can effortlessly bind to tumor cells via the naturally occurring transferrin receptor 1 (TfR1) in cancer cells, generated as an effect of disordered iron metabolism homeostasis, leading to its endocytosis for targeted delivery. Hence, in contrast to conventionally fabricated nanoparticles, the synthesized ferritin nanoconjugate system offers several advantages, including intrinsic tumor-targeting ability, favorable pharmacokinetics and safety profiles, excellent antitumor activity, easy scaling up and manufacturing with robust and reproducible procedures, and a universal drug-loading platform.^7^ These unique properties make ferritin nanoconjugates an ideal vehicle for efficient anticancer drug delivery.

Furthermore, in order to reduce the emergence of resistance and improve the disease-free survival system, it is of utmost importance to conquer improved effectiveness of the currently available therapeutic agents.^8^ This can be achieved either by modifying the already available drugs structurally, hence generating their derivatives, or by presenting the combination therapy idea with existing drug agents to attain the synergistic effect of each drug. Till date, plentiful agents have been explored to be used in combination with prevailing therapeutics. To this approach, an FDA-approved compound valproic acid (VPA) has been exposed as a histone deacetylase (HDAC) inhibitor, to work efficiently in cancers both in preclinical and clinical settings.^9^ It has reported efficiencies in altering the proliferation, growth, migration, as well as hormone receptor expression in different cancer types, particularly breast cancers.^10^ However, VPA has majorly been proclaimed as an adjunctive agent to repurpose its use, in combination with many renowned cytotoxic, immunotherapeutic, and hormonal agents to cure cancers.^11^ Likewise, among the various anticancer drugs in the market, doxorubicin (Dox) has been profoundly used in clinics due to its dominant therapeutic effects on an extensive range of cancers.^12^ Yet, the cytotoxic effect of Dox is not limited to only cancer cells, which in turn brings a large number of side effects in vivo.^13^ To overcome this challenge, the encapsulation of Dox in a coating material, e.g., liposome,^14^ or incorporating it along with a carrier protein and PLGA nanoparticles has been fairly tested in the medicinal field.^15^

Herein, our encapsulating drug of choice was valproic acid, mainly to question its suitability only as an adjuvant therapy. Consequently, its nanoconjugate, i.e., ferritin-VPA, was designed to observe the exclusive effect of VPA when given through some carrier. Second, for a better comparison, another nanoconjugate was designed, viz. ferritin-VPA-Dox, to further inquire into the adjuvant effect of VPA with some known anticancer drug (for instance, Dox), when given through a carrier (ferritin) molecule. Interestingly, our newly devised nanoconjugates allowed the delivery of substantial doses of VPA and Dox to tumor cells devoid of the need for supplementary ligand modifications or carrier characteristic adjustment. F-VPA and F-VPA-Dox exhibited longer median survival times and lower toxicity when administered at the same dose in all tumor models studied.

Additionally, in the present study, we also set out to validate the diffusion capabilities of ferritin nanoconjugates into the physiologically relevant 3D spheroids using various cancer cell lines to generate spheroid models, thereby transporting the drugs across intact spheroid. To the best of our knowledge, no data have been presented so far to introduce VPA in circulation (alone or as a dual drug delivery payload) along with a carrier protein to observe its improved efficiency or long-term circulation time. Remarkably, within the tumor environment, the oxidative damage caused by VPA and/or VPA-Dox results in the production of reactive oxygen species (ROS).^16^ However, the presence of ferritin increases the intracellular iron concentration, thus promoting ferroptosis (a recently identified, iron-dependent cell death),^17^ which ultimately depends upon the accumulation of iron and lipid ROS. Combining all these effects, these ferritin–drug nanoconjugates show great potential in tumor cell killing. Hence, the outcomes of this ferritin–drug nanoconjugate could pave the way for developing an approach to predict the fate of drug nanoconjugates toward clinical translation.

## Results and Discussion

### Designing the Protein–Drug Nanoconjugates: a Vision

As a backbone of the study, we desired to synthesize a bionanocarrier-based drug nanoconjugate to fulfill the dire need for an enhanced therapeutic approach in cancer treatment. For this reason, out of the limited list of bionanocarriers, ferritin is as an ideal, naturally occurring carrier candidate that can increase payload stability both in vitro and in vivo, prevent undesirable interactions, and prolong the circulation half-life of small molecules.^18−20^ However, to fulfill the drug of choice given through a carrier molecule, valproic acid (VPA) was picked first because VPA itself has little side effects, and second, it exhibits a broad anticancer effect in various cancers despite having a limited therapeutic efficacy of its own.^11^ Attributed to this reason, previously, it has been stated that VPA is generally used as an anticancer adjuvant drug or as a combination therapy with other antitumorigenic agents (e.g., Dox/PTX, etc.) and immunotherapeutic and hormonal agents.^21^

Therefore, to utilize the therapeutic potential of VPA at its fullest, with a longer availability of the drug in circulation, we hypothesized the utilization of VPA alone as an effective yet exclusive tumor therapy while delivered via some carrier molecule, in particular, a protein. To the best of our knowledge, none of the studies so far have investigated testing VPA alone delivered through a protein carrier. Keeping this concern in mind, we utilized ferritin to first design ferritin-VPA (F-VPA) nanoconjugates, benefiting from ferritin’s structural properties as a nanocarrier for small molecules.^22^ Second, in order to have a comprehensive investigation output, we also desired to testify the coloading strategy of drugs while given via a carrier molecule, to identify the synergistic, additive, and/or antagonistic behavior of VPA in conjugation with some renowned anticancer agent as this has also not been reported yet. For this purpose, we designed another nanoconjugate, i.e., ferritin-VPA-doxorubicin (F-VPA-Dox), with a proposition to observe a meaningful difference from the available scientific outcomes on VPA drug combinations, without carriers.

Remarkably, to our interest, out of the two designed nanoconjugate variants (F-VPA and F-VPA-Dox), a significantly enhanced effect of VPA (with a proportion of approximately 3-fold) has been observed when given through a carrier (i.e., F-VPA nanoconjugate) as compared to VPA alone (further detailed analysis and interpretations in the proceeding sections), thus highlighting the prominence of nanocarriers in the targeted drug delivery approach.

Interestingly, the second designed nanoconjugate, viz. F-VPA-Dox, also showed a much prominent synergistic effect of both drugs (VPA and Dox) when given through a biocarrier molecule (ferritin), irrespective of these drugs when given alone (data given in the following sections), thereby emphasizing the effective outcome of our given hypothesis and proving the concept of nanocarrier-based therapies as effective regime in cancer treatment and cure.

### Ferritin: a Universal Drug-Loading Platform

Ferritin, being a protein biomolecule, possesses multiple noncovalent as well as covalent interactions (e.g., hydrogen bonding, ionic interactions, hydrophobic interactions, van der Wall forces, as well as disulfide bonding) due to the presence of a variety of amino acids building its complex structures. Thus, the loading and/or entrapment of any foreign molecule into the ferritin cage is highly dependent on these interactions. So far, a large number of external molecules have been productively encapsulated into the ferritin structure,^23^ signifying ferritin as a universal cargo carrier for tumor-targeted delivery. For instance, it has been used for the entrapment of both metal- and nonmetal-based anticancer agents, where binding of the common anticancer drug doxorubicin to ferritin nanocages is mainly reliant on electrostatic interactions. Since the p*K*_a_ of Dox is 8.2,^24^ it can effortlessly bind to the internal negatively charged ferritin nanocage surfaces in neutral buffering conditions.^25^ Consequently, it can be anticipated that under neutral aqueous conditions, any small molecule as a chemotherapeutic drug that can attain positively charged groups can be entrapped into the cavity of ferritin. Some more of positively charged drugs entrapped into ferritin include cisplatin and some of its derivatives, most of alkylating agents,^26^ and a number of antibiotics other than Dox (such as daunorubicin, idarubicin),^27^ and vinblastine (a plant alkaloid),^28^ among others.

### One-Step Synthesis and Biophysical Characterization of Ferritin–Drug Nanoconjugates

**Scheme 1:**
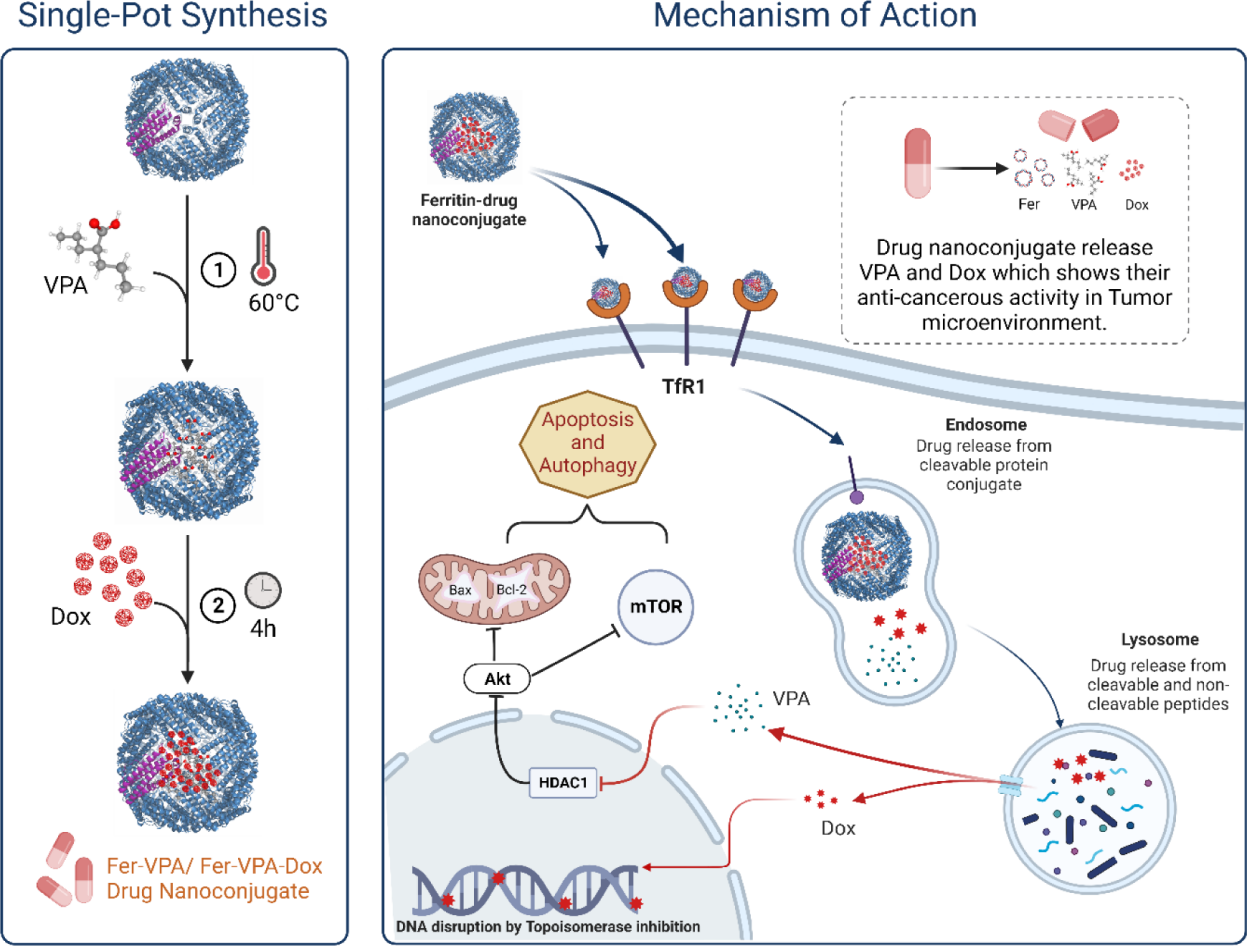
Route of Protein–Drug Nanoconjugate Synthesis Using Thermal-Induced Switch of the Drug Entry Channel in Ferritin Nanocages and Tumor Killing Mechanism

**Figure 1:**
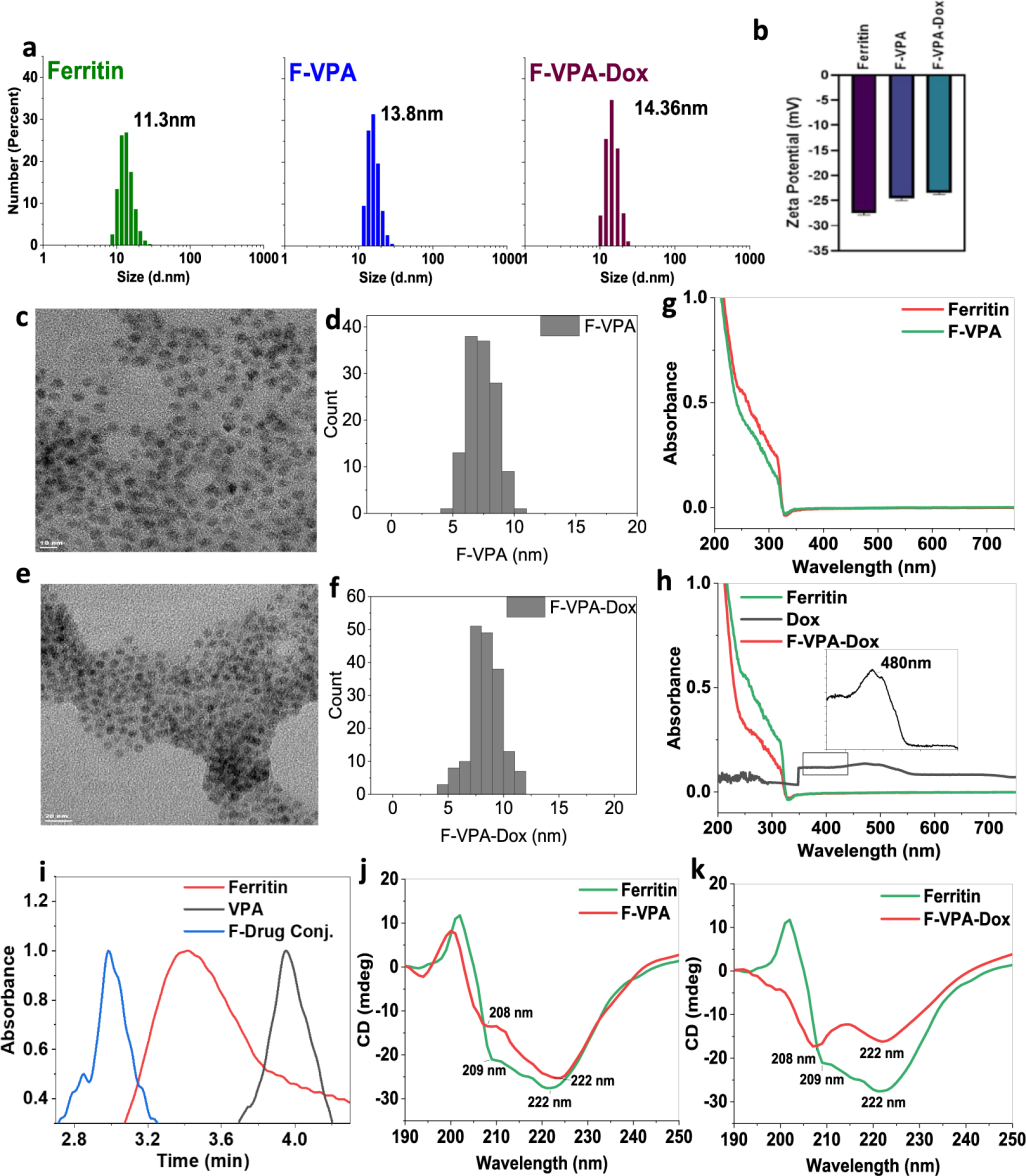
Characterization of F-VPA and F-VPA-Dox nanoconjugates. (a) Size distribution and (b) zeta potential of ferritin alone, F-VPA, and F-VPA-Dox determined by DLS, (c) TEM image of the synthesized nanoconjugate F-VPA, (d) the mean graphical size obtained from TEM images using the ImageJ software for F-VPA and (e,f) likewise for F-VPA-Dox, respectively, (g) UV–visible spectrophotometric analysis of F-VPA and (h) F-VPA-Dox, (i) size exclusion chromatography (SEC) to confirm the drug conjugation with protein (ferritin), and (j,k) circular dichroism (CD) spectroscopic analysis of ferritin alone, F-VPA, and F-VPA-Dox nanoconjugates.

Ferritin existence as a naturally available protein reduces the immunogenicity concerns with an additional manipulation of payload at the cancer site via the enhanced permeability retention effect.^29^ Thus, the encapsulation of VPA and Dox in ferritin cages leverages nanoconjugates for improved chemotherapy. The ferritin nanoconjugates (F-VPA/F-VPA-Dox) comprising ferritin, valproic acid, and/or doxorubicin were synthesized using the channel-based loading strategy for numerous drug molecule encapsulation.^30^ It has been reported that the pH-based loading method denatures the ferritin molecule by damaging the amino acid residues in the protein cage, thus disrupting the stability of the synthesized drug nanoconjugates. To overcome this challenge, we decided to stick to the channel-based drug loading approach for F-VPA/F-VPA-Dox nanoconjugate production (Figure S1), which enhanced drug release effectiveness in acidic circumstances. The fabrication process is illustrated in Scheme 1, highlighting the thermoresponsive synthesis of protein–drug nanoconjugates, where the natural entry channel of ferritin protein opens by increased temperature, which can expand the size of the channel for drug entry, and thus successfully loads Dox and VPA. Following the synthesis, the hydrodynamic size of F-VPA and F-VPA-Dox nanoconjugates was determined via dynamic light scattering (DLS), which indicated monodispersed morphology of the obtained nanoconjugates. Figure 1a illustrates an average diameter of 11.3 nm for ferritin alone. However, the harvested protein–drug nanoconjugates showed an average diameter of 13.8 and 14.3 nm for F-VPA and F-VPA-Dox, respectively, which is closer to the bare-native protein, thus, confirming the synthesis of drug-loaded protein nanoconjugates. These findings also proposed that ferritin remained monodispersed and in a single protein form and did not agglomerate after drug loading. Our findings are coherent with the previously reported data,^31^ thereby suggesting a model size of biocompatible nanocarriers ideal for the enhanced permeability and retention (EPR) effect. Additionally, the zeta potential observations exposed a single population both for ferritin alone (−27.8 mV) and ferritin–drug nanoconjugates as −28.3 mV for F-VPA and −24.9 ± 1.8 mV for F-VPA-Dox (Figure 1b), thereby not showing any significant difference at pH 7, leading to the consideration that the drugs have not just adsorbed nonspecifically onto ferritin shell but rather encapsulated in the ferritin nanocage.^32^ Likewise, the negative surface potential of ferritin–drug nanoconjugates would also contribute to the stability as well as shielded transport in the bloodstream.

Following the size, the morphological analysis of ferritin–drug nanoconjugates was demonstrated by TEM, where the images revealed a monodisperse, sphere-like morphology of the synthesized nanoconjugates with an average uniform diameter of ∼7.5 and ∼8.6 nm, further complementing the size data of F-VPA (Figure 1c,d) and F-VPA-Dox obtained from DLS (Figures 1e,f and S2).

The UV–vis spectroscopic analyses show the peak for bare protein at 280 nm; a standard peak value for proteins,^33^ with a slight variation at around 278 nm when observed for F-VPA nanoconjugates (Figure 1g), depicting the successful formation of the drug–protein nanoconjugate without altering the native structure of the protein. Comparatively, the peak absorption for F-VPA-Dox between 350 and 800 nm increases evidently compared with that of ferritin and/or F-VPA, knowing that Dox shows its respective absorbance under the UV spectrum at around 480 nm (Figures 1h and S3). The results are in line with previous findings presenting ferritin nanoparticles with other drug types.^33^

Furthermore, the encapsulation of VPA and Dox by ferritin was further analyzed on an Agilent Technologies (1200 series) HPLC system using a Superdex 200 10/300 GL gel filtration column, with the running buffer (100 mM PBS, pH 7.2) on size exclusion chromatography (SEC). SEC analyses of ferritin before and after VPA and Dox encapsulation were monitored at 280 nm. The column calibration was followed using the same buffer conditions before running the samples. According to SEC elution results, the elution time of F-VPA/Dox nanoconjugates was slightly earlier in comparison to ferritin and drugs alone (before encapsulation), indicating that a slight increment in the size of ferritin was acquired after drug conjugation (Figure 1i). These results are agreeably complementing the DLS and TEM data of ferritin–drug nanoconjugates reported in the preceding results section (Figure 1a,c,e) as well as comparable to the ferritin-loaded paclitaxel nanoparticles reported in earlier studies.^34^ Summarizing the basic characterizations, a well-defined nanosized ferritin-based drug nanoconjugate was ready for further evaluation and testing on 3D cancer spheroid models.

### Structural Insights into Protein–Drug Nanoconjugates

Circular dichroism (CD) spectroscopy was used as an investigative tool in order to look into the secondary structural alterations in ferritin with respect to VPA and Dox interactions. CD spectroscopy can analyze the conformational changes and biochemical implications of native proteins and any alterations in protein structure due to external factors, for instance, pH, temperature, surfactants, etc., and/or conjugation with drug molecules. It has been demonstrated that the secondary structure of ferritin comprises α-helix with a typical peak pattern of minima at 222 and 208 nm and a maximum at 198 nm,^35^ when measured in the far-UV region (190–250 nm) of CD spectroscopy.

Since, in our study, the nanoconjugates were synthesized via heating-based passive loading of drug (VPA/Dox) molecules within the ferritin cage, CD spectra were recorded for ferritin both in the absence and presence of drugs (Figure 1j,k) to essentially analyze if the ferritin structure remains stable after thermal exposure and after drug conjugation. Interestingly, the CD spectrum of ferritin–drug nanoconjugates (F-VPA/F-VPA-Dox) also showed the same minimum peaks around 222 and 209 nm as that of ferritin alone,^36^ confirming the persistence of mainly α-helical structure with a trivial reduction in the ellipticity. This can be allied by the interference of drugs (in particular, Dox) with the supportive bonding forces and structural interactions of the protein, resulting in structural transitions and hence alteration in the native protein (ferritin) structure. Consequently, the thermal encapsulation of the drug all in all did not affect the structure of the ferritin cage, resulting in a stable and biocompatible nanoconjugate availability for drug delivery.^37^

### Drug Entrapment into Ferritin and In Vitro Drug Release

**Figure 2:**
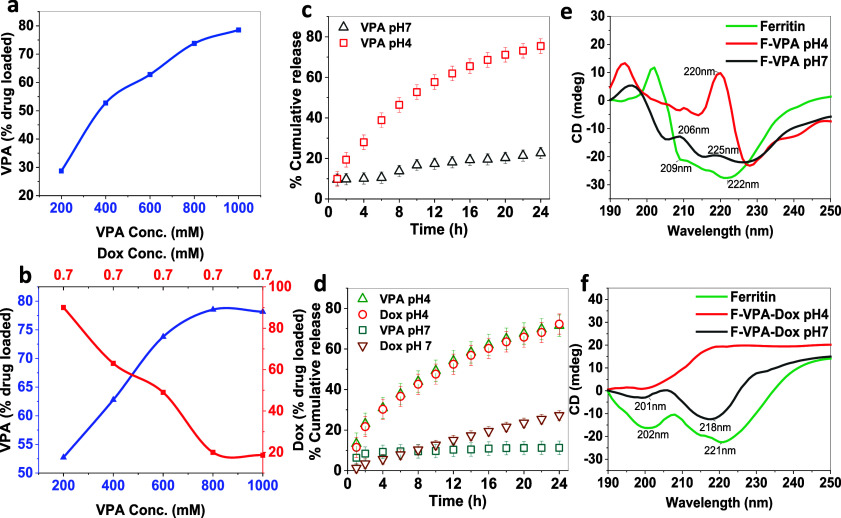
(a) Drug loading of VPA in F-VPA at increasing concentrations, (b) drug loading of VPA at an increasing concentration (0.2–1 mM) and Dox at a constant concentration of 0.7 mM in F-VPA-Dox, (c) in vitro release analysis of the F-VPA nanoconjugate in PBS (pH 7) and glycine buffer (pH 4) over the period of 24 h, (d) cumulative drug release of VPA and Dox from the F-VPA-Dox nanoconjugate at different pH values, (e) circular dichroism indicating structure alteration of ferritin to release VPA in neutral to acidic conditions, and (f) structure alteration in ferritin to release VPA and Dox at different pH values indicated from CD.

Ferritin is a large protein with a large ability to passively load drug molecules inside. Elevating the temperature facilitates the opening of channels within the ferritin structure, enabling the passage of small molecule drugs into the ferritin cage. Consequently, drugs can be effectively loaded into ferritin nanocages through this process. Notably, doxorubicin (Dox), epirubicin, cisplatin, and oxaliplatin have been successfully loaded into ferritin cages using heat-induced passive diffusion.^38^ Therefore, at first, the drug-loading capacity of ferritin (Figure 2) was optimized by a series of VPA concentrations, i.e., 0.2, 0.4, 0.6, 0.8, and 1.0 M for the F-VPA nanoconjugate (Figure 2a), whereas for F-VPA-Dox, the dual drug-loading ability was estimated by using the same increasing VPA concentrations (0.2–1.0 M) while keeping the Dox concentration constant at 0.7 mM during this nanoconjugate formation (Figure 2b). It was evident from the results that increasing concentrations of VPA led to higher drug loading in ferritin molecules, with the highest loading efficiency at 1.0 M (1000 mM), i.e., 80% (Figure 2a). However, interestingly, the drug-loading ability of VPA varied greatly with the dual conjugation of the second drug, i.e., Dox. Since at lower doses of VPA, the binding sites are in abundance, which gives more room for Dox binding, with increasing concentrations of VPA and competition of binding sites between the two drugs, the maximum loading ability of VPA was observed at 0.8 M (800 mM), i.e., 75%, while the Dox-loading concentration was 60% at maximum with 0.7 mM (Figure 2b).

Studies have also shown that most of the proteins can reversibly disassemble into protein subunits under acidic conditions; therefore, they can be used as drug carriers to encapsulate and hence release the encaged drug molecules to the surrounding environment.^39^ Ferritin, being a strong bioinorganic protein, exhibits remarkable resistance to extreme environmental conditions, for instance, elevated temperatures (∼85 °C) and alkaline pH levels (8.5–9) for a definite period, without substantial disruption of its intricate quaternary structure. This structure originates from the self-assembly of 24 polypeptide subunits organized into a hollow sphere with an internal diameter of 8 nm.^40^ Studies by Sato et al. exhibited the impact of pH on the structural regulation of ferritin, highlighting that ferritin, at an acidic pH, can dissociate into dimers while maintaining the native-like structures (at both secondary and tertiary levels).^41^ However, with the increase in pH to neutral or slightly basic values (pH 7.2–8.0), it can reassemble back to the original 24-mer ferritin. Additionally, other factors, including ionic strength, also impact the reassembling rate of ferritin, signifying that between the assembly units, the localized, predominantly repulsive, electrostatic interactions positively influence the kinetics of ferritin self-assembly.^42^ Interestingly, manipulating pH and ionic strength offers a method to encapsulate small molecules within ferritin nanocages, presenting opportunities for various applications like drug delivery.

Keeping in mind the pH-sensitive depolymerization characteristics of ferritin, we investigated the pH-dependent drug (VPA/Dox) release kinetics from ferritin–drug nanoconjugates (i.e., F-VPA/F-VPA-Dox) by simulating the endolysosomal and physiological conditions.^43^ For this purpose, PBS (pH 7) and glycine–HCl buffer (pH 4.0) were used to mimic the physiological and endolysosomal conditions, respectively, and ferritin nanoconjugate constructs were incubated in the prepared buffer for more than 24 h to monitor the release profiles of VPA and Dox from the ferritin nanoconjugates (Figure S4). In order to calculate the drug release, MS-QToF was used to estimate the relative abundance of both drugs (Figure S5), released individually in the buffer medium.

As hypothesized, it was found that at an ambient temperature, ferritin–drug nanoconjugates (i.e., F-VPA/F-VPA-Dox) were quite stable at pH 7 with only <22 ± 3.6055% release of VPA from the F-VPA conjugate after 24 h of incubation. While for the other ferritin nanoconjugate construct, i.e., F-VPA-Dox, the release of both drugs was studied simultaneously, at predesignated time intervals. Here, as a synergistic, dual drug delivery approach, only a minimal amount of VPA release was observed, i.e., around 11 ± 3.282% after 24 h, whereas Dox release was approximately 25 ± 2.3574%. This can be attributed to the nonspecific and/or competitive binding of both drugs and slight adsorption of Dox molecules on the ferritin cage.^44^

Contrary to this, when the pH of the surrounding buffer medium was decreased to an acidic environment (i.e., pH 4), due to the predicted protein dissociation, the drugs (VPA and Dox) from ferritin–drug nanoconjugates exhibited rapid release as compared to pH 7. For instance, the liberation percentage of VPA loaded in the F-VPA conjugate was up to 77 ± 2.7735% after 24 h (Figure 2c,d), whereas >72 ± 5.2696% and 79 ± 4.9923% release of both VPA and Dox, respectively, was monitored at the acidic pH after 24 h (Figure 2c,d). These findings propose that a maximum release of Dox and VPA is conceivable once the ferritin cages are disassembled inside the cell by endosomal and lysosomal ingestion, at an acidic pH 4.0, also validated by the available literature.^7^ The results confirmed that the ferritin nanoconjugate constructs are not stable at an acidic pH, thus releasing Dox and VPA in endolysosomal acidic conditions after being encapsulated by cancer cells.^45^

The result indicates that the drugs (VPA/Dox) were appropriately entrapped in the ferritin cage and were well protected with a negligible burst release. These findings also clearly validate that the release of the drug cannot be triggered unless a required stimulus (pH) is provided, and since ferritin nanoconjugate constructs are not stable at an acidic pH, they release the drug in endolysosomal acidic conditions after being ingested into the tumor environment.^46^

The release data of ferritin nanoconjugates were further supported by CD spectroscopy, at both pH 7 and 4 (Figure 2e,f). The resulting CD spectrum provides insights into the overall folding and structural features of ferritin. Given the presence of dominant α-helical structure in globular ferritin protein, typically showing the characteristic patterns corresponding to these structural elements, we postulated that ferritin possesses the ability to encapsulate the drugs (VPA/Dox) through noncovalent interactions like electrostatic interactions, hydrogen bonding, and hydrophobic interactions, utilizing amine groups that can form electrostatic interactions with charged residues on the surface of ferritin and/or hydrophobic regions of drugs that can interact with specific amino acid residues on the surface of ferritin.

In our observations, it is noteworthy that the α-helical pattern of F-VPA/F-VPA-Dox did not alter under neutral (pH 7) conditions (Figure 2e,f), displaying the typical peak minimum pattern of ferritin, with only a slight variation in both magnitude and shape of CD bands due to the presence of small molecules in the structure,^47^ hence confirming the intact structural behavior of ferritin at this pH range, as discussed earlier. However, at pH 4, the structure of ferritin nanoconjugates (F-VPA/F-VPA-Dox) showed complete disruption with a significant loss in the ellipticity values at 222 and 208 nm, indicating the opening of the ferritin α-helix structure and thus providing drug release at an acidic pH. These spectra also revealed that due to the denaturation of ferritin at an acidic pH, the entrapped drug molecules can be effortlessly released into the tumor acidic microenvironment.

### Biocompatibility of Ferritin–Drug Nanoconjugates Using Hemolysis Assay: an Ex Vivo Study

**Figure 3:**
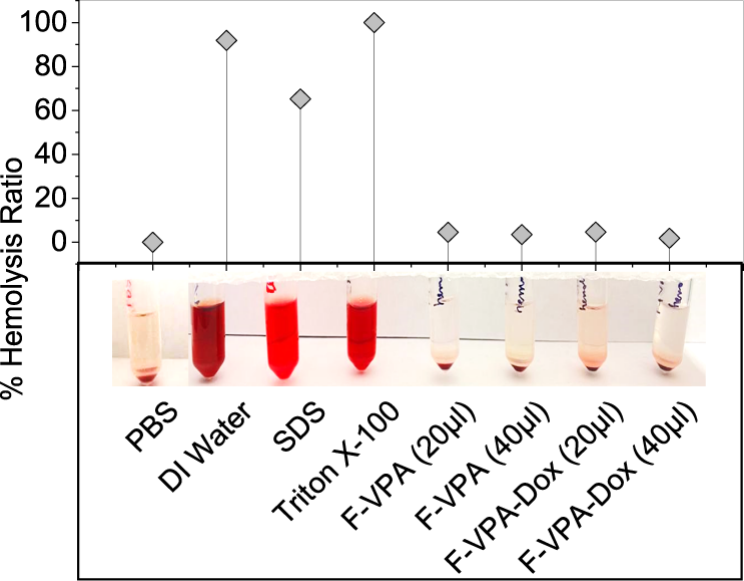
Hemolysis test of ferritin–drug nanoconjugates. Images of centrifuged RBC solutions and the percentage hemolysis of F-VPA/F-VPA-Dox after incubation with different concentrations of nanoconjugates. PBS was used as negative control, whereas Triton X-100 and deionized water were used as positive controls.

To evaluate the biosafety of our synthesized ferritin–drug nanoconjugates, we conducted hemolysis and cytotoxicity analyses using respective assays. The images of RBC solutions (centrifuged) and hemolysis ratios are presented in Figure 3. Studies ascertain that the encapsulation of drugs (Dox and/or VPA) in a biocompatible protein structure as a drug carrier can greatly improve the drug conjugate behavior in the biological environment.^48^ Thus, for the hemolytic activities of the ferritin nanoconjugates, two concentrations of F-VPA and F-VPA-Dox, i.e., at a volume of 20 and 40 μL (in 1 mL of 2% RBC suspension) were nominated as tests. Moreover, DI water, SDS, and Triton X-100 were used as positive controls, and PBS was used as a negative control. The absorbance results showed that F-VPA and F-VPA-Dox produced almost no hemolysis (i.e., <2%) of red blood cells even at higher concentrations, irrespective of 48 h of incubation, which indicated that the ferritin–drug nanoconjugates can be safe and biocompatible to be used as an intravenous (IV) injectable drug.^49^

### Tumor Regression Studies of Ferritin–Drug Nanoconjugates

#### Cancer Cells Targeting of Ferritin–Drug Nanoconjugates

The interaction among ferritin and tumor cells has been testified to be facilitated by transferrin receptors (TfR1), present on the majority of cancer cell lines. We conducted cell viability studies on MCF-7, C4-2, and HT-29 cells. These cell lines were specifically selected based on their increased TfR1 expression as it promotes ferritin binding and thus can enhance the cellular uptake of ferritin-based carrier therapeutic alternatives by cancer cells.^50,51^ Thus, we started to evaluate the cytotoxicity profiles of our designed ferritin nanoconjugates F-VPA and F-VPA-Dox for their anticancerous efficacy. As positive controls, the drug alone (VPA and Dox) as well as the native ferritin variant were used for efficacy comparison with the nanoconjugates.

#### Optimization of Nanoconjugate Drug Doses Using 2D Monolayer Cells

**Figure 4:**
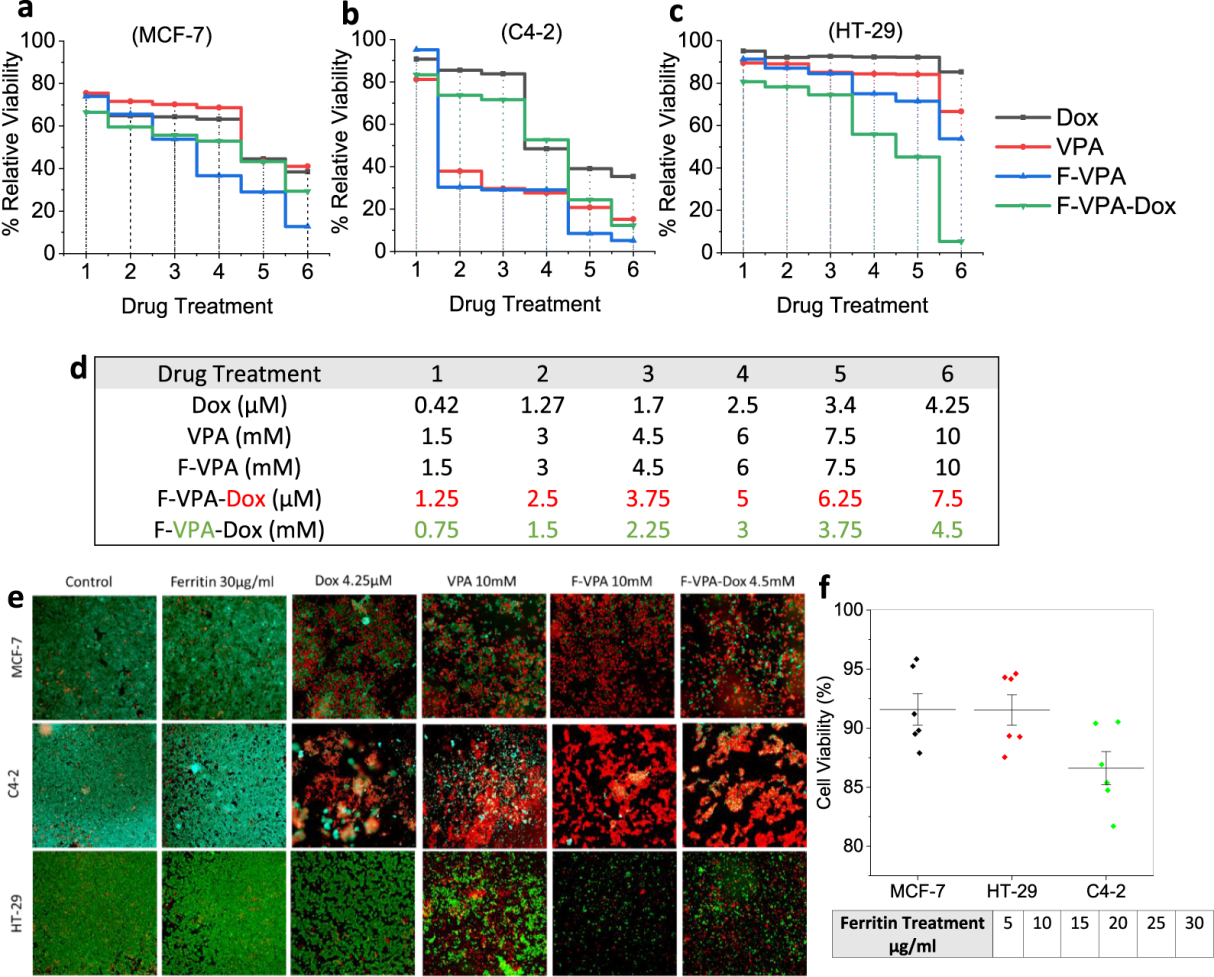
Optimization of nanoconjugate drug doses (F-VPA and F-VPA-Dox) on 2D monolayer cancer models (a) MCF-7, (b) C4-2, and (c) HT-29, (d) with increasing concentrations. (e) Live–dead cell-stained images with Calcein-AM (green) and PI (red) showing live cells in green and dead cells in red after drug conjugate treatment. (f) Testing the interference of ferritin as a carrier protein with cytotoxic behavior of drug in increasing concentrations.

We preliminarily optimized the possible drug concentrations and doses after treating 2D monolayers of each cell line used (MCF-7, C4-2, and HT-29) with a series of controls and corresponding nanoconjugate concentrations, such as 1.5, 3.0, 4.5, 6.0, 7.5, and 10 mM for VPA in the F-VPA nanoconjugate, whereas 0.75, 1.5, 2.25, 3, 3.75, and 4.5 mM for VPA, and 1.25, 2.5, 3.75, 5.0, 6.25, and 7.5 μM for Dox in the F-VPA-Dox nanoconjugate, as shown in Figure 4, showing the concentration-dependent effect on monolayers.

As anticipated, out of the various concentrations tested, each cancer cell line behaved differently when treated with both F-VPA and F-VPA-Dox nanoconjugates, thus first emphasizing the organ-to-organ variation in cancer cells and correspondingly their altered response to the same treatment.^52^ For instance, the results for the breast cancer cell line (MCF-7) in Figures 4a,e and S6A showed that the F-VPA conjugate has an IC_50_ value at around 4.5 mM, whereas the corresponding VPA alone doses were not sufficient to show similar cytotoxicity even at a 6 mM equivalent dose, thus showing the enhanced efficacy of the F-VPA nanoconjugate when given as a nanocarrier version because of cell cycle arrest and apoptosis induced by VPA.^21^

Likewise, F-VPA-Dox showed a synergistic therapy effect, giving IC_50_ cell viability even at 2.25 mM VPA doses with a corresponding Dox concentration of 3.75 μM, where controls did not show efficacy even at higher doses, i.e., VPA 6 mM and Dox 4.25 μM. Herein, the synergistic behavior of both drugs, i.e., VPA and Dox, was determined via the Chou–Talalay analysis^54^ to calculate the combination index (CI) (Table S1), which is a widely accepted method for determining drug interactions, where a CI value of <1 would indicate synergy, a value equal to 1 would suggest an additive effect, and a value >1 would suggest antagonism. Using the values from Figure 4a for the 2D cell culture experiments of the MCF-7 cell line, the calculated CI was found to be 0.02%. Consequently, any CI value below 1 is considered synergistic, with lower values indicating a stronger synergy between the drugs. The obtained results hence confirm the effect of VPA-Dox to be strongly synergistic when given through a carrier protein ferritin. There are several proposed mechanisms involved in the efficacy of Dox as an anticancerous drug, which includetumor suppressor gene p21 expression, p53-dependent cell cycle arrest, and increase in oxygen radicals, resulting in apoptosis. Notably, the delivery method of Dox greatly influences the Dox activity against cancer. For instance, a high-dose bolus injection leads to apoptosis associated with G2 arrest and high expression of BAX and p21.^53^ However, cancer cells exposed to a constant level of Dox concentration show remarkably low apoptosis, thus impacting the potential therapeutic response of Dox alone. Saha et al.^54^ revealed that the combined therapy of 5 mM VPA and 250 nM Dox generated great synergistic potential in hepato-carcinoma via ROS generation and autophagy. Moreover, VPA enhances the internalization of Dox molecules in cancer cells.^54^ However, lack of a drug carrier makes this therapy insufficient for clinical applications.

Moving to another cancer type, i.e., prostate cancer (C4-2) (Figure 4b,e), the cells showed a significant decline in cell viability when treated with F-VPA even at 3 mM doses, when the corresponding equivalent control dose of VPA alone (6 mM) showed less efficacy in comparison. This can be attributed to the sustained VPA release from ferritin nanocages, which decreases cell viability through cell cycle arrest, p21 protein expression, and apoptosis, resulting in decreased telomerase activity in cancer cells.^10^

Whereas, justifying our second designed nanoconjugate, i.e., F-VPA-Dox, it explains that in recent research, the combination treatments for cancers with conventional drugs such as Dox, sorafenib, and cisplatin show a limited response rate (approximately 15–20%) when tested on various cancer types. However, considering the synergistic approach of combination drugs with a CI value of 0.16% (Table S1), in our case, VPA is reported to sensitize the cancer cells for enhanced internalization of Dox via the caveolae-mediated endocytosis pathway, leading to more profound and targeted results when given via a carrier protein. Our study revealed that 3.75 mM VPA and 3.75 μM Dox from the F-VPA-Dox bioconjugate were enough for the cell viability to reach much below IC_50_ values, whereas alone drugs, even at 6 mM VPA and 2.5 μM Dox, were unable to achieve similar IC_50_ values for cell viability.

Last, the results were less pronounced in colon cancer (HT-29) (Figures 4c,e and S6B) as compared to the other two cell lines of different cancer types. Here, the VPA and Dox controls did not show any significant cell death even at higher concentrations of 10 mM and 4.25 μM, respectively. However, with F-VPA conjugation, we observed that 50% cell viability was achieved at 10 mM, whereas for F-VPA-Dox, a synergistic effect produced cell death even at 2.25 mM VPA and 3.75 μM Dox concentrations, with an obtained CI value of 0.31 (Table S1). The resistance can be attributed to the reduction of HDAC2 expression level that plays an essential role in colorectal carcinoma response to DNA-damaging agents alone or in combination.^55^ Here, the combined treatment effect was compared with a single treatment alone. All combined treatments tested were found to exert a synergistic effect on cell death in HT-29 cells as compared with alone treatments.

Also, to confirm if the carrier is interfering with the cytotoxicity behavior of drugs, when applied as its nanoconjugate counterparts, ferritin alone was tested at varying concentrations from 5 to 30 μg/mL (Figure 4f,e) in all three cancer cell lines, i.e., MCF-7, HT-29, and C4-2, and did not show any interference and/or toxicity to cancer cells.

#### Three-Dimensional (3D) Spheroid Modeling

A growing body of research indicates that primary cells maintained in three-dimensional (3D) cultures show physiological characteristics longer than those generated in a two-dimensional (2D) culture. One reason for this stays as even while immortal cells undergo transformations and could have several chromosomal abnormalities, it seems that they can regain some of their physiological characteristics when cultivated in three dimensions, perhaps going back to their original differentiation route.^56^ Second, the unpredictability and intricacy of the extracellular matrix (ECM) present significant obstacles for the administration of drugs and the customization of nanomedicine penetrations.

**Figure 5:**
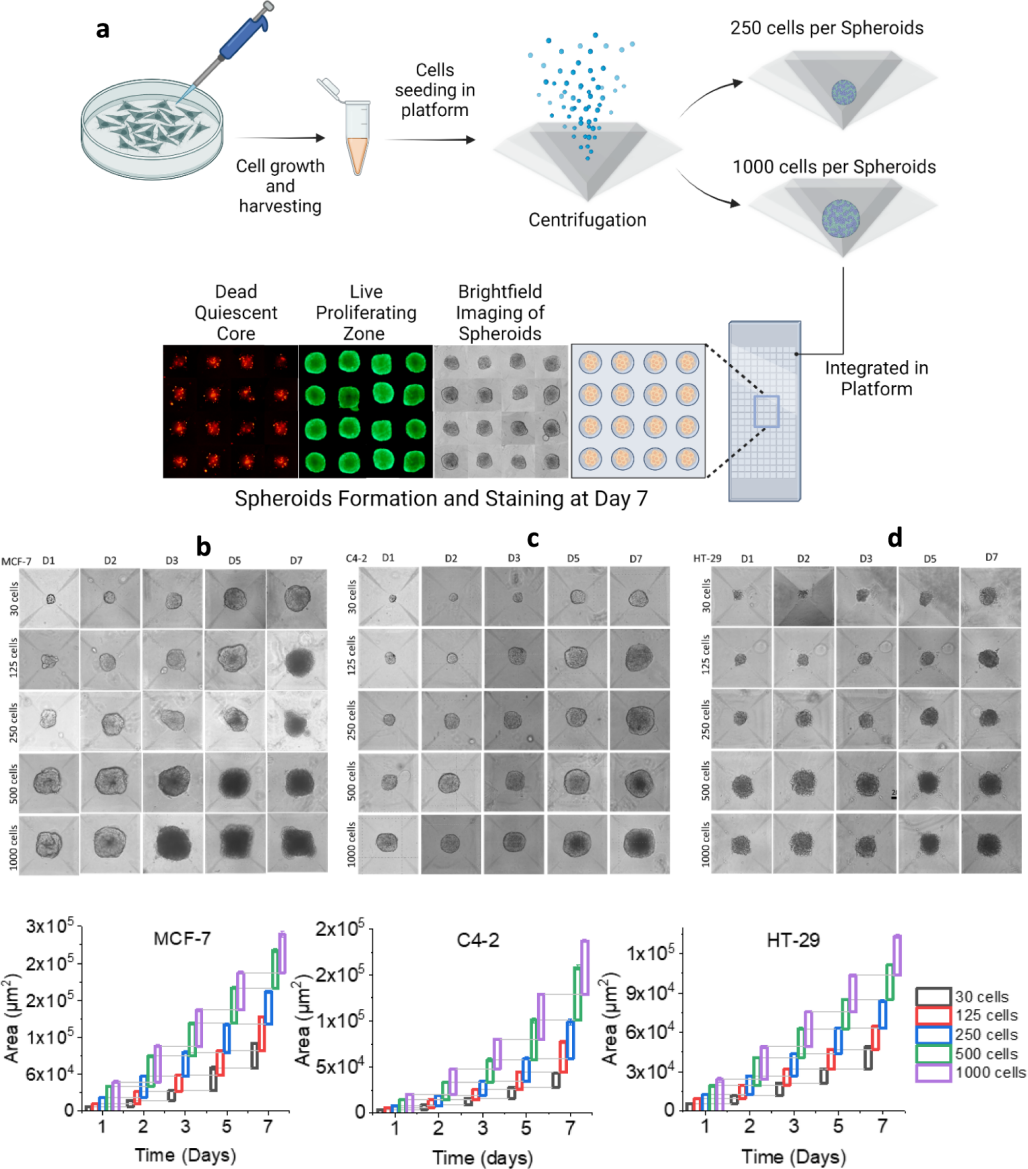
(a) Schematic of 3D spheroid formation depicting the layout from cell seeding until spheroid formation using customized microwell platforms. (b–d) Optimization of spheroid growth and size with respect to varying cell number seeded for each cell line.

However, in order to attain highly efficient and controllable tumor models in vitro, there is a dire need to generate a 3D model system out of various cancer cell lines that offers a state-of-the-art growth pattern monitoring for each cell line as a benchmark setup. Therefore, prior to experimentation with ferritin drug nanoconjugates, multiple cell lines, including MCF-7, a breast cancer cell line; C4-2, a prostate cancer cell line; and HT-29, a human colon cancer cell line, were characterized for their spheroid modeling and growth rate with respect to their seeding cell number and volume. The schematic of spheroid formation (Figure 5a) and an actual picture of generated spheroids are shown in Figure 5b. The spheroid culture was seeded in a standardized 96-well plate containing a customized μ-well platform. The seeded cells got aggregated at the bottom of an individual μ-well and eventually formed spheroids under defined medium conditions. Figure 5a (schematic) displays in detail the complete formation of spheroids after 7 days of incubation, further stained for live/dead cell imaging. Calcein-AM indicated live cells (>90%)in the proliferating layer at the outer edges, while an inner necrotic zone of dead cells was in prominent red when stained with PI, indicating a hypoxic region with dead cells. It is of high significance since a substantial rise in TfR1 receptors is induced under in vitro hypoxic circumstances, indicating a potential function for TfR1 in tumor angiogenesis.^57^

Additionally, another major factor intertwined with the state-of-the-art benchmark setup of spheroidal modeling is the “attained size” with respect to its growth behavior, corresponding to their cell seeding intensity. This phenomenon also carries greater significance for the optimization of desired spheroid volume, paving the way for highly controlled and optimized drug treatment analysis. Therefore, we first conducted the size monitoring experiment related to cell seeding number, as shown in Figure 5b–d. The results provided a very meaningful data, illustrating that although the proliferation of spheroids continued to over a 7-day culture period, the cell packing densities of the various cell lines varied drastically. Despite this, a volume of approximately 300–400 μm was determined to be the “ideal spheroid size” since bigger spheroids (>350 μm) result in an increased number of quiescent cells and a greater core of necrotic cells. It is evident from Videos S1–S3 that the cell packing densities of the various cell lines vary, and this is known to alter the effect of treatment therapies as well.^58,59^ For instance, as seen in the spheroid formation images as well as graphical illustration of each cell line used, MCF-7 cells with 1000 cells/μ-well generated highly packed spheroids (Figure 5b), while C4–2 cells, on the other hand, were able to keep their proliferating zone intact even after 7 days with a steady growth pattern using the same number of seeded cells (Figure 5c). Likewise, HT-29 cells depicted highly packed cell density spheroids with similar numbers of cells seeded (Figure 5d). Consequently, it is evident from the literature that the packing density of tumor cells alter the penetration and efficacy of drug nanoparticles and conjugate,^58,59^ and this is known to affect how effective certain therapies can be and considered to be one of the major advantages of the 3D spheroid models over the 2D monolayer models, i.e., its ability to replicate this in vivo impact.^60^ In view of the above understanding, VPA- and Dox-encapsulated ferritin nanoconjugates were therefore tested for tumor penetration using multicellular cancer spheroid (MCS) models.

#### Antitumor Drug Treatment Efficacy of Ferritin–Drug Nanoconjugates on 3D Spheroids

Based on the 2D monolayer results as well as 3D spheroid modeling data, the study was carried forward to its actual scope of testing the designed ferritin nanoconjugates on physiologically relevant 3D spheroid models to understand and hence to a certain degree relate the drug nanoconjugate response to the tumors when present in vivo. For this, the optimized drug concentrations from 2D experiments were shortlisted to be 6 mM F-VPA and 3 mM F-VPA-Dox, as a double-dose regime, alongside the corresponding doses of the drug alone (for both VPA and Dox) selected for the treatment of 3D spheroids of the respective cell lines. Thus, in total, a concentration of 12 mM F-VPA and 6 mM F-VPA-Dox in two consecutive doses separated by a time period of 24 h was applied. At the end of the experiment, spheroids were trypsinized and stained for live (Calcein-AM) and dead cell (PI) stains.

**Figure 6:**
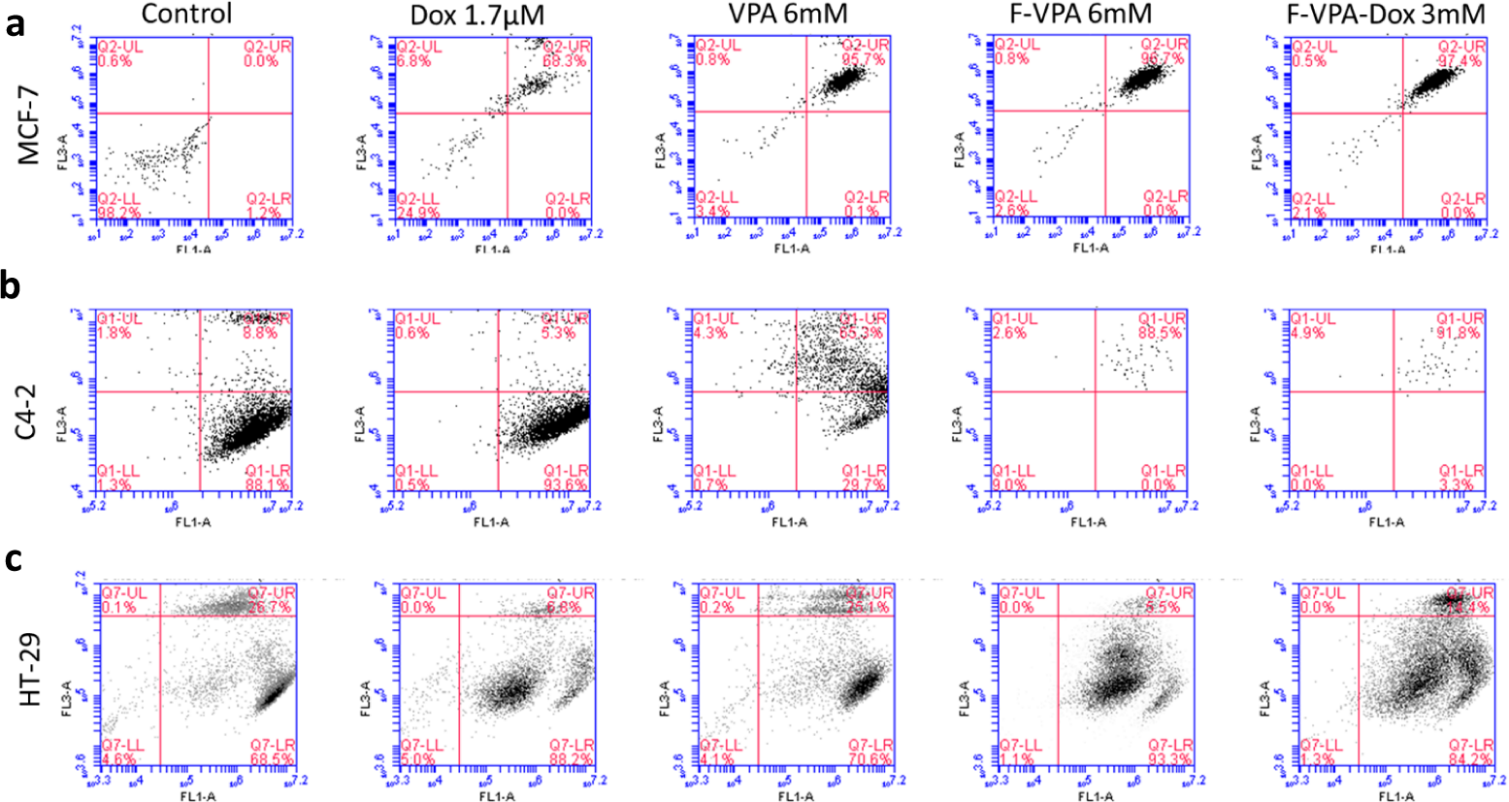
Flow cytometric analysis of viable cells in 3D spheroid models after treatment with drug nanoconjugates for F-VPA and F-VPA-Dox in comparison to alone drug controls (Dox and VPA) for (a) MCF-7, (b) C4-2, and (c) HT-29.

The flow cytometry viability analysis of spheroids cultured and treated for 4 days was observed to analyze the drug treatment efficacy. The left panel of the figure shows the forward-scattered light (FSC) and side-scattered light (SSC) plot with gating to avoid the undesired noise from debris. The results in Figure 6 showed that selected doses were effective for cell death in 3D spheroids in all cell lines, with a slight variation in the viability outcomes. For instance, MCF-7 spheroids (Figure 6a) showed the live cell percentage to be 98% in the control (with no drug treatment), whereas for F-VPA- and F-VPA-Dox-treated spheroids, the viable cell percentages dropped to 2.6% and 2.1%, respectively. In prostate cancer (C4-2) (Figure 6b), we observed that the viable cells were 93.6% in the control, while treated cells’ viability was only 9% and 3% in F-VPA and F-VPA-Dox, respectively. Interestingly, for HT-29 spheroids (Figure 6c), the live cell count was comparatively less for F-VPA treatment as compared to F-VPA-Dox, i.e., 8.5% and 11.4%, respectively, whereas for the control, it was 26%. This can be attributed to the resistance of the Dox drug due to the reduction of Dox accumulation ability in the nucleus and thus affecting the downstream events in colon spheroids that form a more physiologically relevant model.^61^

Overall, in the 3D spheroidal analysis, the increased resistance and hence high-dosage application of the ferritin nanoconjugates to each cell line are expected due to a more realistic tumor-like microenvironment introduced by 3D spheroids. This is one of the several reasons that 3D spheroids are advanced, closely mimicking preferable models over to 2D monolayers for drug testing and hence have been shown in several spheroid model studies conducted with conventional drugs, including Dox.^1,62^ Digging more into the theoretical understanding and relevance, it is predicted that due to the introduction of 3D cellular interaction, a modified extracellular matrix (ECM) environment is produced by cancer cells, which in turn interrupts drug penetration.^63^

#### Microscopic Analysis for the Selective Uptake of Ferritin–Drug Nanoconjugates by 3D Spheroids

**Figure 7:**
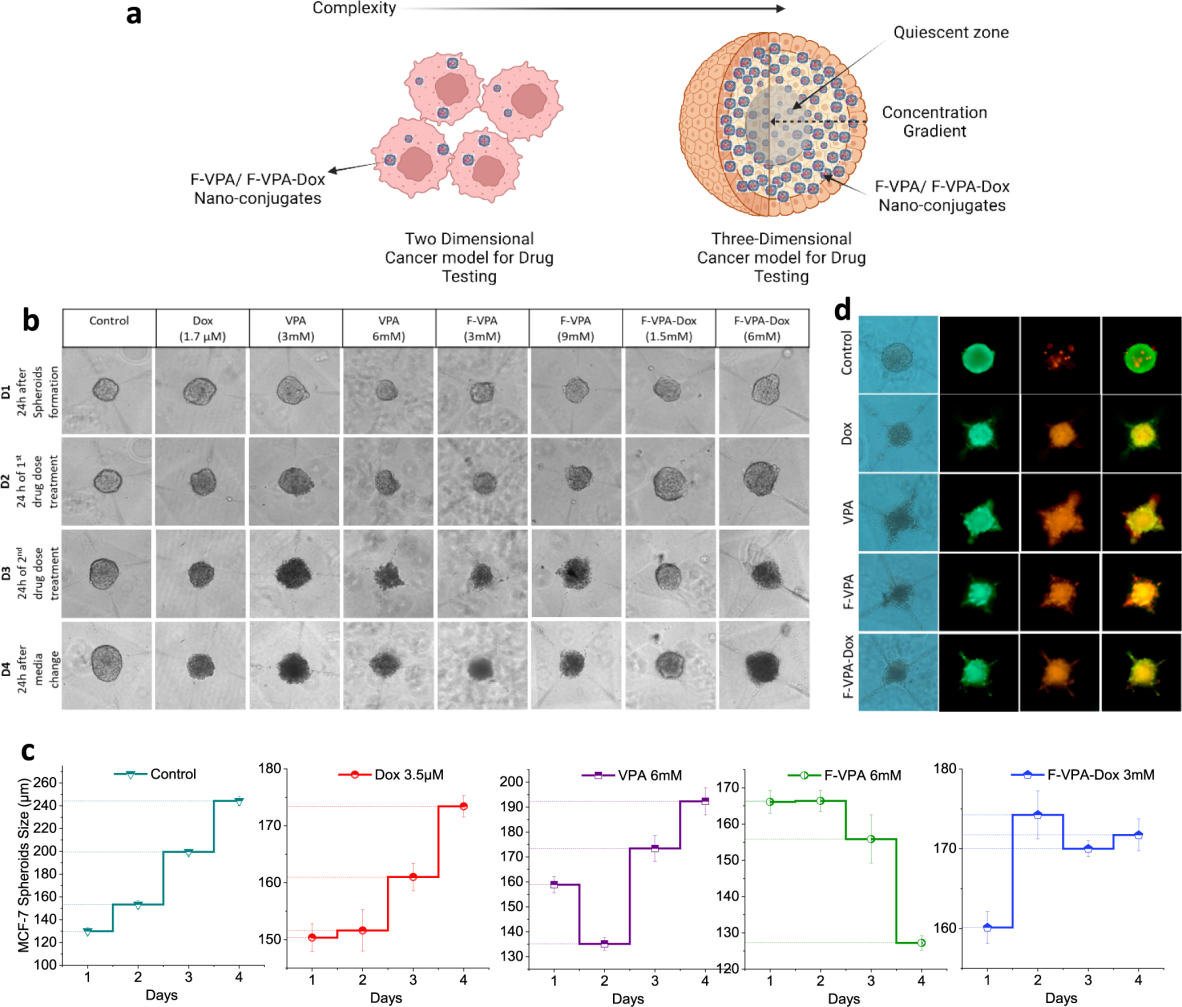
Drug treatment on 3D spheroid models. (a) Schematics of a comparison between 2D monolayer and 3D spheroid model with altered drug penetration to the deep tumor regions. (b) Bright-field imaging of MCF-7 spheroids for 4 consecutive days after drug treatment. (c) Graphical representation of drug effects on spheroid size over the period of 4 days. (d) Live–dead cell-stained images of spheroids at the end of drug treatment showing live cells in the periphery and dead cells in the core.

A quick translation to commercial therapeutics can be achieved by introducing a more complicated 3D tumor spheroid model, which can better replicate the complex environment present in vivo than a 2D monolayer. The presence of a hypoxic necrotic region in the core and a proliferating cell layer outside makes spheroids more realistic tumor model for drug testing, as shown in the schematic (Figure 7a). Thus, to verify the effect of the defined doses on cancer 3D spheroid models, the spheroids were fabricated and observed initially for their proliferation and growth using bright-field imaging. When spheroids reached a definite size of approximately 150 μm, the respective doses of drug alone (as controls) and ferritin nanoconjugates (as treatment groups) were administered. The spheroids were then observed for 72 h (i.e., 3 consecutive days) for morphology and growth behavior. The results showed a subsequent decrease of up to 50 ± 10 μm in spheroid size with a significant difference in the morphology after drug nanoconjugate treatment, and the dead cell count increased within two consecutive doses of both variants of nanoconjugates (F-VPA and F-VPA-Dox) in all cell lines. Unlike bare drugs, the ferritin protein nanoconjugates have the ability to penetrate via TfR1 receptors and thus disrupt the spheroid structure, resulting in the ECM disorganization produced by cell–cell interactions and thereby promoting the penetration of F-VPA and F-VPA-Dox near the core region of spheroids and giving an efficient drug response of either VPA and/or VPA-Dox.

Elaborating on the findings of each cell line one by one in detail, in MCF-7-treated cells (Figure 7b), it has been observed that F-VPA at a working dose of 6 mM and F-VPA-Dox at a dose of 3 mM decrease cell viability and disrupt the proliferating zone completely, as evident from the spheroids’ size reduction to 128 and 170 μm, respectively, at day 4 as compared to the control size reaching 240 μm in the same time (Figure 7c). The spheroids were stained at day 4 after treatment, showing increased necrotic regions and depletion of the proliferative zone, as shown in treated spheroids (Figure 7d). This enhanced effect is due to ferritin binding to TfR1 receptors and VPA causing downregulation of the antiapoptotic regulating factor Bcl-2, thus increasing cell death and proliferation.^64^ The results show that the synthesized nanoconjugate has the potential to show improved therapeutic response when administered through a carrier-based targeted delivery approach.

**Figure 8:**
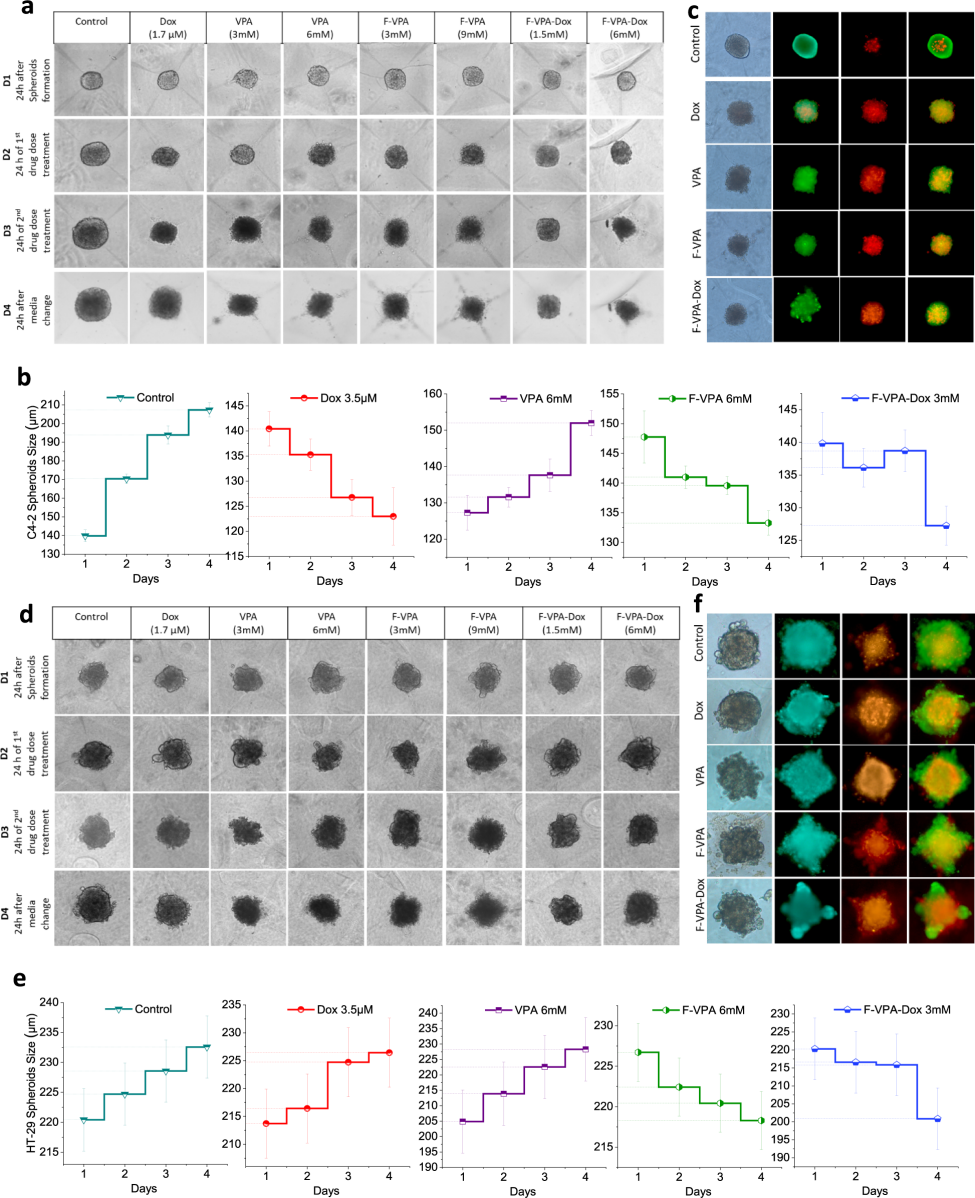
Bright-field imaging of (a) C4-2 and (d) HT-29 spheroids for 4 consecutive days after drug treatment. (c,f) Live–dead cell- stained images of spheroids at the end of drug treatment showing live cells in the periphery and dead cells in the core; and (b,e) graphical representation of drug effects on spheroid size over the period of 4 days.

Similar results were observed in C4-2 cancer cell spheroids for their growth behavior after treatment under bright-field imaging (Figure 8a), which also showed decreased spheroid growth and increased cell death. It was observed that the F-VPA disrupted spheroid organization and halted spheroid growth to 133 μm, whereas for F-VPA-Dox, the size was further reduced to 127 μm in comparison to the control, which continued proliferating to approximately 210 μm in size (Figure 8b) and further stained for live–dead cell imaging in spheroids after treatment (Figure 8c). It has been observed that C4-2 spheroids stopped proliferating after 72 h of two-dose drug regimes. Previous analyses of histone acetyl transferases and histone deacetylases (HDAC) have shown apoptosis as the major mechanism of cell death in prostate cancer.^65^

Moving further, for HT-29 (colon cancer) spheroids, cells are denser and more resistant to treatment even in 2D monolayers; thus, it is expected to have a very high dose of drug nanoconjugate to generate the desired effect on 3D spheroid models. Surprisingly, it was observed that a 9 mM dose of F-VPA and 6 mM dose of F-VPA-Dox were sufficient to stop the growth of spheroids and cause cell death, as observed from the microscopic analysis of spheroid size. The results showed that the spheroid growth was halted at 218 μm after treatment with 3 mM F-VPA, whereas for F-VPA-Dox spheroid, reduction at 200 μm was observed on day 4 (Figure 8d). The control spheroids, however, continued to proliferate up to ∼233 μm in size. Thus, a relatively different behavior was observed with HT-29 spheroids (Figure 8d) when treated with either of the ferritin–drug nanoconjugates (F-VPA/F-VPA-Dox). Theoretically, HT-29 shows transferrin receptor-mediated uptake of VPA, causing an enhanced uptake combined with HDAC inhibition in the nucleus for enhanced apoptosis.^66^ Consequently, even at low doses such as 6 mM of F-VPA, the drug nanoconjugate was sufficient to show a response in spheroid size reduction to approximately 200 μm from the control size of 233 μm, while the alone drug could not halt spheroid growth even at higher doses (Figure 8e). Herein, Dox molecules disrupt the DNA structure and stop the growth and proliferation of cancer cells, as observed in the case of F-VPA-Dox, thus providing a synergistic and pronounced effect of drug conjugates. The stained images after treatment show that the proliferating zone got distinctly disrupted after treatment with conjugates, and the disruption is more pronounced in F-VPA-Dox treatment (Figure 8f).^67^

Notably, in our experimental setup, day 0 was referred to as the first day of drug addition when spheroids reach the desirable size (approximately 200 ± 20 μm). On the other hand, drug alone (Dox and VPA) as controls at similar doses show a moderate effect on the cancer cell spheroids but do not cause complete cell death, allowing the exploration and hence validation of the synergistic and target-specific treatment with ferritin-encapsulated nanoconjugates.

## Conclusions

Taken together, our designed ferritin–drug nanoconjugates (F-VPA/F-VPA-Dox) possess numerous essential characteristics that are advantageous for transformation and translation into clinical tumor therapy. In terms of choice of carrier biomolecule, primarily due to its natural existence in living systems, ferritin-based nanoconjugates hold key amino acids that prevent the activation of immunological and/or inflammatory responses. In addition, due to its structural possessions, the ferritin molecule also allows high loading efficiency for multiple drugs, thereby allowing synergistic therapy as well as controlled drug release properties. Hence, the designed nanoconjugates possess tremendous potential for killing and degradation of cancer cells along with TfR1-mediated endocytosis, which is a useful cascade for drug-selective delivery.

To comprehend these milestones, a couple of essential developments have been achieved. At first, a nanocarrier design concept for VPA was attended to, considering that VPA is generally used as an anticancer adjuvant drug or as a combination therapy with other antitumorigenic agents. Consequently, as a first of its kind, herein, the F-VPA nanoconjugate allowed the successful utilization of the therapeutic potential of VPA at its fullest, providing longer availability of the drug in circulation and offering more than 5-fold increased intratumoral drug concentration compared to free VPA, thereby significantly suppressing tumor growth after a single-dose treatment. Inclusively, the outcome proclaims VPA alone as an effective yet exclusive tumor therapy when delivered via some carrier molecule, in particular, a protein.

Second, in order to have a comprehensive investigation output, the coloading strategy of the drugs was also testified while given via a carrier molecule. F-VPA-Dox served as a meaningful candidate to observe the synergistic behavior of VPA in conjugation with some renowned anticancer agents, which has also not been reported yet, with a proposition to observe significant differences from the available scientific outcomes on VPA-drug combinations without carriers. Overall, the outcome of this current research is influential, disclosing a highly useful all-biocompatible protein nanocarrier, with significant integration of VPA alone as an active component or in combination with Dox. It is acknowledged that our study has tested these ferritin–drug nanoconjugates currently on three different cancer cell lines, which may not fully represent the broad spectrum of cancers. While our findings are promising (in varying extents) in these models, in order to hold the potential to serve as a therapeutic approach toward a broad range of cancers, further investigation across a wider variety of cancer cell types can elaborate further to generalize the efficacy and therapeutic potential of these F-VPA and F-VPA-Dox nanoconjugates. Moreover, the study also needs expansion to evaluate the biocompatibility and potential off-target effects of these nanoconjugates in normal cells, such as fibroblasts or epithelial cells, to ensure the nanoconjugates’ broader biocompatibility.

## Experimental Section

### Materials and Chemicals

Ferritin (F), valproic acid (VPA), and dialysis membrane (12–14 kDa) were acquired from Sigma-Aldrich. Doxorubicin hydrochloride (Dox) was obtained from Biosynth (Staad, Switzerland). Phosphate-buffered saline (PBS) was purchased from Serana (Pessin, Germany). Milli-Q water was used in all the procedures extracted from Merck Milli-Q Direct. All of the supplementary reagents were of analytical grade reagent quality.

### Synthesis of Ferritin–Drug Nanoconjugates

For the synthesis of F-VPA/F-VPA-Dox nanoconjugates, a simple, thermal-responsive, channel-based, drug-loading approach was followed^30^ to load the drug of choice (VPA and/or Dox) onto the protein. For F-VPA synthesis, ferritin (1 mg) was first dissolved in 20 mM Tris–HCl buffer (pH 8.0) until completely mixed. The sample was then incubated at 60 °C, and to this, 1 mL of valproic acid (1 M dissolved in 20 mM Tris–HCl buffer) was added and left at constant mixing for 4 h in order to get the maximum binding of VPA to ferritin. Once completed, the sample was left at room temperature to sustain the native nanocage structure of the protein, now holding the drug, followed by centrifugation at 12,000 rpm/10 min to separate the undissolved material from the supernatant.

Parallel to this, the F-VPA-Dox nanoconjugate was also synthesized using a similar protocol, with the addition of Dox freshly solubilized in water to a final concentration of 1 mg and mixed for 30 min, after VPA in the prepared solution. Once the synthesis was completed, the protein–drug nanoconjugates were dialyzed against water using an MWCO 12–14 kDa membrane (Sigma-Aldrich) to remove free VPA and/or Dox. The loaded VPA and Dox were quantified using a QToF-MS (Agilent Technologies, 6530, Accurate-Mass Q-TOF LC/MS) against the drug alone of similar concentrations.

### Loading/Encapsulation Efficiency

The VPA and Dox/VPA loading rate in weight percent (Dox/ferritin weight %) was calculated using peak abundance values obtained from QToF-MS. The loading capacity (*N*) was calculated as follows:
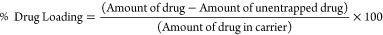

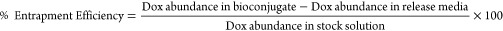


where MW is the molecular weight.

### Characterization of Ferritin–Drug Nanoconjugates

The particle radius, monodispersity, size distribution, and potential analysis of F-VPA and F-VPA-Dox were conducted by a Zetasizer Nano ZSE (Malvern Instruments, Ltd.). The absorption pattern of the samples before and after nanoconjugate synthesis was observed using a protocol previously defined by Munir et al.^68,69^ Briefly, a full scan between 200 and 700 nm wavelength using a UV–vis spectrophotometer (Cary 100 Bio) was conducted against water as a blank as well as a reference solvent. High-performance liquid chromatography–size exclusion chromatography (HPLC–SEC) was performed to measure and further confirm the synthesis of protein–drug nanoconjugates with respect to size variability using Agilent Technologies (1200 series) equipped with a 2998 photodiode array detector.

In order to observe the morphology of F-VPA and F-VPA-Dox nanoconjugates, transmission electron microscopy (TEM, Tecnai G2, FEI) was used. For sample preparation, 2 μL of the samples before and after nanoconjugate synthesis was dropped onto a Formvar-Carbon-coated 400 mesh copper TEM grid (Agar Scientific, UK) at room temperature. After being completely dried, the samples were imaged with an FEI Tecnai TF20 FEG high-resolution TEM, operating at 200 kV. To calculate the average size and standard deviation of protein–drug nanoconjugates, an image processing tool (ImageJ 1.50i) was used to calculate the average of 50 particles in the TEM image.

The particle size and surface charge of F-VPA and F-VPA-Dox nanoconjugates were measured in aqueous solution on a Zetasizer Nano ZS (Malvern Instruments Ltd., MA, USA), with a 633 nm wavelength laser light at a scattering angle of 173° and 90° collecting optics. For each measurement, 0.3 mL of the sample was poured into the disposable DLS and capillary zeta potential cuvettes, and observation was made in the automatic mode. Data were analyzed using the Malvern Dispersion Technology software 7.0.2.

### Circular Dichroism Study

The analysis of the secondary structure of ferritin before and after binding with the drugs (VPA/Dox) was conducted through CD spectroscopy using a protocol previously defined by Munir et al.^68^ Briefly, a Jasco J-815 CD spectrometer (Model 150-S) was used, and for each sample, measurements were obtained in triplicate in the far-UV range (190–250 nm), with a 1 nm bandwidth and 1 nm step size. The spectra were smoothed by the Savitzky–Golay method with a polynomial order of 2.

### In Vitro Drug Release

In order to observe the release profile of drugs (VPA/Dox) from F-VPA and F-VPA-Dox nanoconjugates, a dialysis process was implemented, as done by Nazir et al.^69^ Briefly, 0.1 M PBS (pH 7) and glycine–HCl buffer (pH 4.0) were used as the release meda. Concentrated samples were poured into the dialysis membrane (MWCO 12–14 kDa), positioned in a beaker containing 15 mL of these buffers under constant magnetic stirring for 24 h. At prearranged time points, 0.5 mL of the medium was taken out and exchanged with fresh PBS (of the respective pH) at equal volume. The drug release was measured by the QToF-MS method against the drug alone of similar concentration. For the Q-TOF MS analysis, 100% pure water (solvent A) was used as the mobile phase at a flow rate of 0.1 mL/min for both VPA and Dox conjugates of ferritin, and the detection was conducted with UV–vis absorption.

### Hemolysis Assay

The hemolysis assay was conducted following the protocol previously defined,^49^ with slight modifications. Briefly, the blood was drawn from a healthy volunteer and poured in an anticoagulant blood collection tube (with heparin). The blood was centrifuged at 2000 rpm for 10 min to separate the serum and acquire red blood cells (RBCs). The supernatant (serum) was removed, and RBCs were washed gently with PBS (three times), followed by a centrifugation step under similar conditions as previously defined. Next, a 2% RBC suspension was prepared using PBS, and different concentrations of F-VPA and F-VPA-Dox nanoconjugates were added in separate tubes. Every test sample was incubated with 1 mL of 2% suspension at 37 °C for 2 h. Samples in DI water and detergents (SDS/Triton-X100) and PBS were taken as positive and negative controls, respectively. At last, the solutions were centrifuged again, and 250 μL of the supernatant was poured into a 96-well plate to measure the absorbance at 545 nm in a microplate reader (BioTek, uQuant, USA). To calculate the hemolysis ratio (%), the following formula was used:
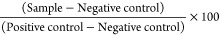
where above represents the absorbance values of the sample, negative control, and positive control, respectively.

### Cell Culture–Standard 2D Culture Conditions

MCF-7, a breast cancer cell line and HT-29, a colon cancer cell line were purchased from AP Institute (Turkey). MCF-7 and HT-29 cells were cultured in Dulbecco’s modified Eagle medium (DMEM, Gibco) supplemented with 10% fetal bovine serum (Serana) and 1% penicillin and streptomycin (Serox), whereas for C4-2, RPMI 1640 with l-glutamine (Serana) supplemented with 10% fetal bovine serum (Serana), 1% penicillin, and streptomycin (Serox) were used. Cells were maintained at 37 °C incubator with 5% CO_2_ within a humid atmosphere. For cell dissociation, 0.2% trypsin–EDTA (Gibco) was used. All cells were grown in T-75 flasks to form a monolayer at approximately 80% confluency. For the 2D monolayer cell model, cells were plated such that the final confluency at the end point is approximately 70% in 96-well plates, with an initial cell count of 500 cells/well. Once plated, cells are left in the incubator at 37 °C and 5% CO_2_ for 24 h to ensure adherence, after which experiments are initiated.

### Drug Treatment and Viability Assessment

For the 2D monolayer cell model, each cell line was plated in 96-well flat-bottom plates, leaving one row for control and treating the rest of the wells with sequentially increased concentrations of Dox (0.42, 1.27, 1.7, 2.5, 3.4, and 4.25 μM), VPA (1.5, 3, 4.5, 6, 7.5, and 10 mM), F-VPA (1.5, 3, 4.5, 6, 7.5, and 10 mM), and F-VPA-Dox (0.75, 1.5, 2.25, 3.0, 3.75, and 4.5 mM) equivalent concentrations of VPA, whereas 1.25, 2.5, 3.75, 5.0, 6.25, and 7.5 μM with respect to equivalent Dox concentrations were added in each row in only one dose once cells reached 75% confluency. Every concentration was added in triplicate on three different plates per cell line. After 48 h of treatment, the media were removed completely and replaced with fresh media. Cells were observed every 24 h using bright-field microscopy with an inverted Zeiss Observer 7 microscope.

### Three-Dimensional (3D) Spheroid Culture Conditions

For 3D spheroid modeling, a customized platform was fabricated in the Yesiloz Research Laboratory at UNAM-Bilkent University. The platform consisting of 20 μ-wells was fixed in a 96-well plate after sterilization and then left in PBS until further use. For spheroid development, cells were dissociated from monolayers using 0.2% trypsin–EDTA (Gibco) and then counted for viable cell numbers using trypan blue solution (Sigma-Aldrich) and a manual Thoma cell counting chamber (Marienfeld cell counter). At cell viability >95%, cells were seeded in spheroid platforms, as shown in Figure 5. For the optimization of spheroid size, a range of cell numbers was seeded to observe their growth and size potential individually. We seeded 30, 125, 250, 500, and 1000 cells/microwell for each cell line and observed them for 7 days under bright-field microscopy. Once the size of each individual cancer cell line was optimized, further drug experiments were performed.

### Drug Treatment and Spheroid Proliferation

The spheroid size needed for the treatment of the drug was optimized to be approximately 150 μm with the seeding cell density set to 800 cells/microwell. Cells were counted and seeded in each well of a 96-well plate consisting of 20 μ-wells. The platform was subjected to centrifugation for 5 min at 1250 RCF (Nuve centrifuge) and then left for spheroid formation in an incubator at 37 °C and 5% CO_2_ for 6 days or until the spheroids achieved an approximate size of 150 μm. Similar to the 2D monolayer treatment, one row was left as a control, and each row on the 96-well plate consisting of the spheroid platform was treated in triplicate with previously optimized doses of Dox (1.7 μM), VPA (3 and 6 mM), F-VPA (3 and 9 mM), and F-VPA-Dox (1.5 and 6 mM) on three different plates for each individual cell line. The first dose was added to the spheroids once the desirable size was achieved, and the second dose was added after 24 h. Then, after 48 h of treatment, the media from the spheroids were replaced with fresh media. The spheroids were observed for 72 h after the first dose treatment using bright-field microscopy.

### Live/Dead Cell Staining for Fluorescence Microscopy

For 2D monolayers, live–dead cell staining was performed after 48 h of the first treatment. The media were removed from each well, and the cells were washed with PBS thrice. Once the cells were washed properly, 20 μM Calcein-AM (live cell) stain was added enough to cover the cells completely, followed by 20 min of incubation. Next, the cells were washed again and stained with propidium iodide (dead cells) stain at a concentration of 1 nM, followed by a subsequent incubation of 2–3 min. The stains were removed completely, and the cells were submerged in PBS for visualization under an inverted Zeiss fluorescent microscope. The stained cells were observed at 490 and 617 nm for Calcein-AM and PI stain, respectively. The images were taken 5× and analyzed further for relative cell viability percentages using the ImageJ software.

For 3D spheroids, the same protocol was followed to stain the live proliferating zone and the dead necrotic zone of spheroids. The stained spheroids were observed at 20× using an inverted fluorescent microscope.

### Flow Cytometry

The spheroids are taken out of the platforms after the treatments in order to perform the flow cytometry study on the drug-treated spheroids. Following harvesting, the spheroids are gently pipetted up and down for 5 min to decrease the aggregated cell population after subjecting them to trypsin–EDTA 0.25% dissociation into single cells. Propidium iodide (PI) and Calcein-AM dyes are employed in the cell viability experiment to label living and dead cells, respectively. Cell-permeable Calcein-AM can be transformed from nonfluorescent to green fluorescent Calcein by hydrolyzing the acetoxymethyl ester using intracellular esterases in living cells. In contrast, PI exhibits a spectral change upon interaction with DNA and is often impermeable to living cells. Therefore, it has been used to label necrotic or late apoptotic or dead cells with damaged cell membranes.

After the spheroids have been extracted, a single cell suspension (1.5 × 10^5^ cells/mL) in a volume of 1 mL is incubated for 20 min at room temperature and shielded from light with 2 μL of live stain, Calcein-AM (50 μM). The suspension is subsequently incubated with 1.5 × 10^5^ cells in 100 μL of Dulbecco’s phosphate-buffered saline (DPBS, Gibco) for 2–3 min in PI 5 μL (dead stain). The stained cells are analyzed by a flow cytometer (BD flow cytometer) using 488 nm excitation and measuring green fluorescence emission for Calcein-AM (530 nm/30 nm band-pass) and red fluorescence emission for PI (617 nm long pass).

### Statistical Analysis

All the experiments were performed in triplicate. All data are presented as “mean ± standard deviation (SD).” An unpaired *t* test or one-way ANOVA was used to assess the statistical significance, where a *p* value <0.05 was considered statistically significant.

## Supporting Information Available

The synthesized protein–drug nanoconjugates F-VPA and F-VPA-Dox using thermal conditions (60 °C for 4 h) (Figure S1); morphological analysis of protein–drug nanoconjugates F-VPA and F-VPA-Dox using TEM (scale bar 10 and 20 nm) (Figure S2); UV–visible spectrophotometric analysis of Dox alone (as a control for F-VPA-Dox) showing peak maxima at 480 nm (Figure S3); sample preparation and collection setup for drug release at different pH conditions (Figure S4); QTOF data of the drugs used for protein–drug nanoconjugate formation (VPA alone showing molecular ion (M^+^) peak at 143 nm; Dox alone showing molecular ion (M^+^) peak at 397 and 544 nm) (Figure S5); detailed 2D monolayer images of cancer cell lines MCF-7 cells and HT-29 cells before and after the treatment of drug alone and protein–drug nanoconjugates (Figure S6); the CI calculated at the indicated concentration of VPA and Dox (Table S1) (PDF)
Monitoring the spheroid growth of C4-2 cells on different days (MP4)
Monitoring the spheroid growth of HT-29 cells on different days (MP4)
Monitoring the spheroid growth of MCF-7 cells on different days (MP4)
