# Supplementary material for: Unlocking Nature’s Potential: Ferritin as a Universal Nanocarrier for Amplified Cancer Therapy Testing via 3D Microtissues
Source: ACS Appl Mater Interfaces. 2024 Dec 11;16(51):70187–204. doi: 10.1021/acsami.4c12524 (PMC11672483; doi:10.1021/acsami.4c12524)
Supplement: Supplementary file 1 — am4c12524_si_001.pdf [file am4c12524_si_001.pdf]

SUPPORTING INFORMATION

Unlocking Nature's Potential: Ferritin as a Universal Nano-Carrier for Amplified Cancer Therapy Testing via 3D-Microtissues

Iqra Munir[a], Faiqa Nazir[a,b] and Gurkan Yesiloz[a,b]*

[a] *National Nanotechnology Research Center (UNAM)- Bilkent University, 06800, Cankaya-Ankara, Türkiye*

[b] *Institute of Material Science and Nanotechnology, Bilkent University, 06800, Cankaya-Ankara, Türkiye.*

**E-mail: gurkan.yesiloz@bilkent.edu.tr*

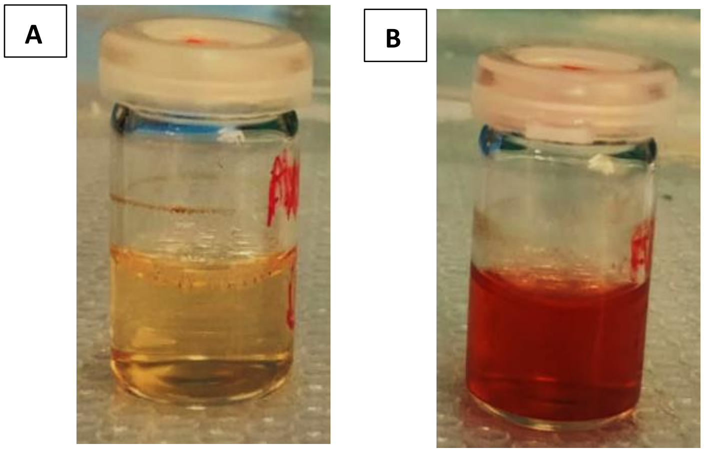

Figure S1. The synthesized protein-drug nanoconjugates (A) F-VPA (B) F-VPA-Dox using thermal conditions (60°C for 4h).

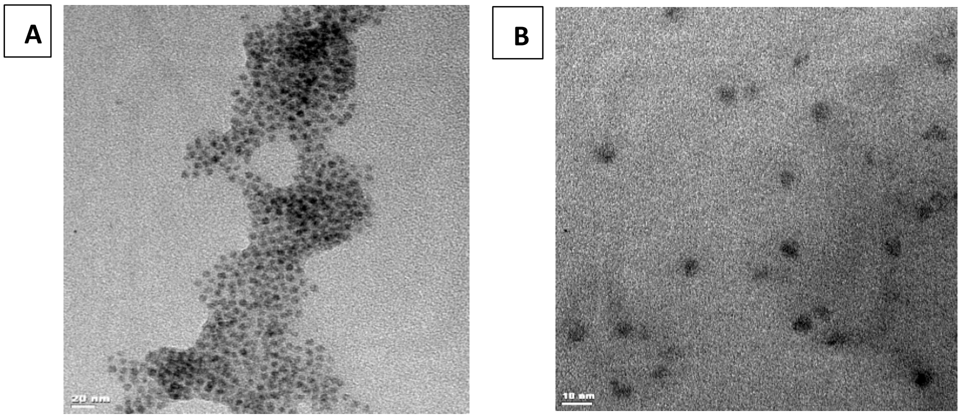

Figure S2. Morphological analysis of protein-drug nanoconjugates (A) F-VPA (B) F-VPA-Dox using TEM (scale bar 10nm and 20nm).

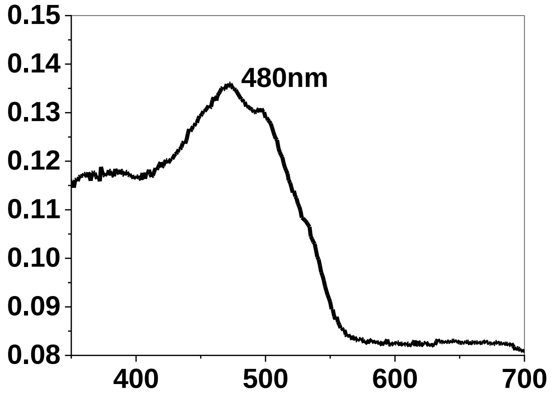

Figure S3. UV-Visible spectrophotometric analysis of Dox alone (as a control for F-VPA-Dox) showing peak maxima at 480nm.

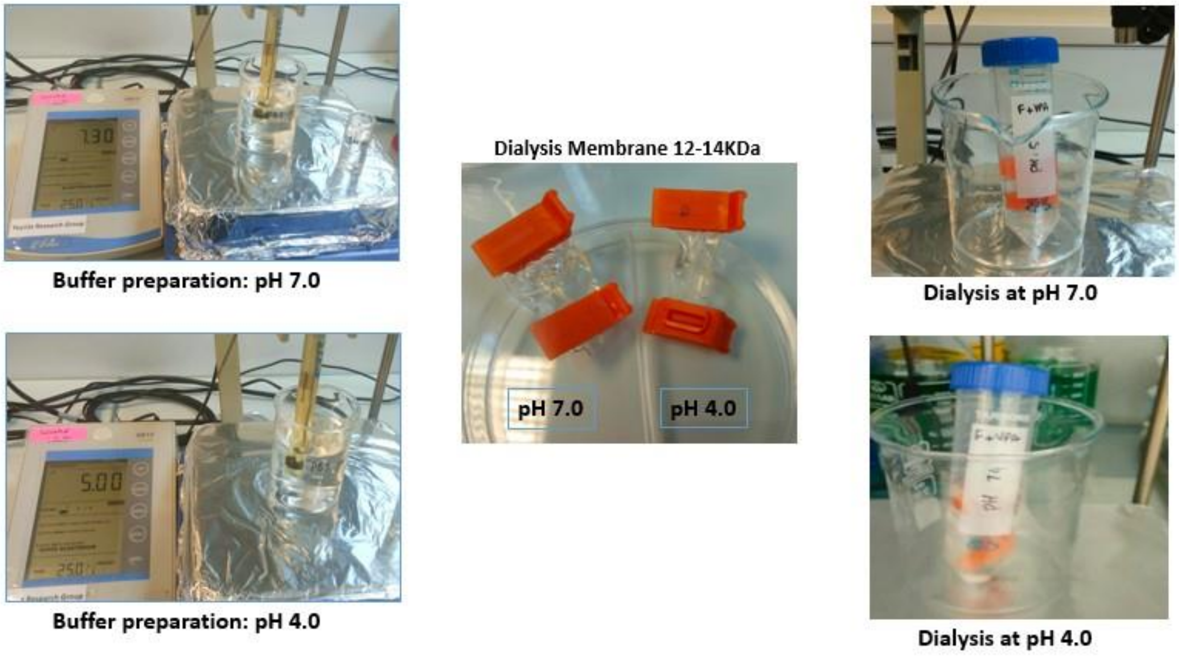

Figure S4. Sample preparation and collection setup for drug release at different pH conditions.

A

B

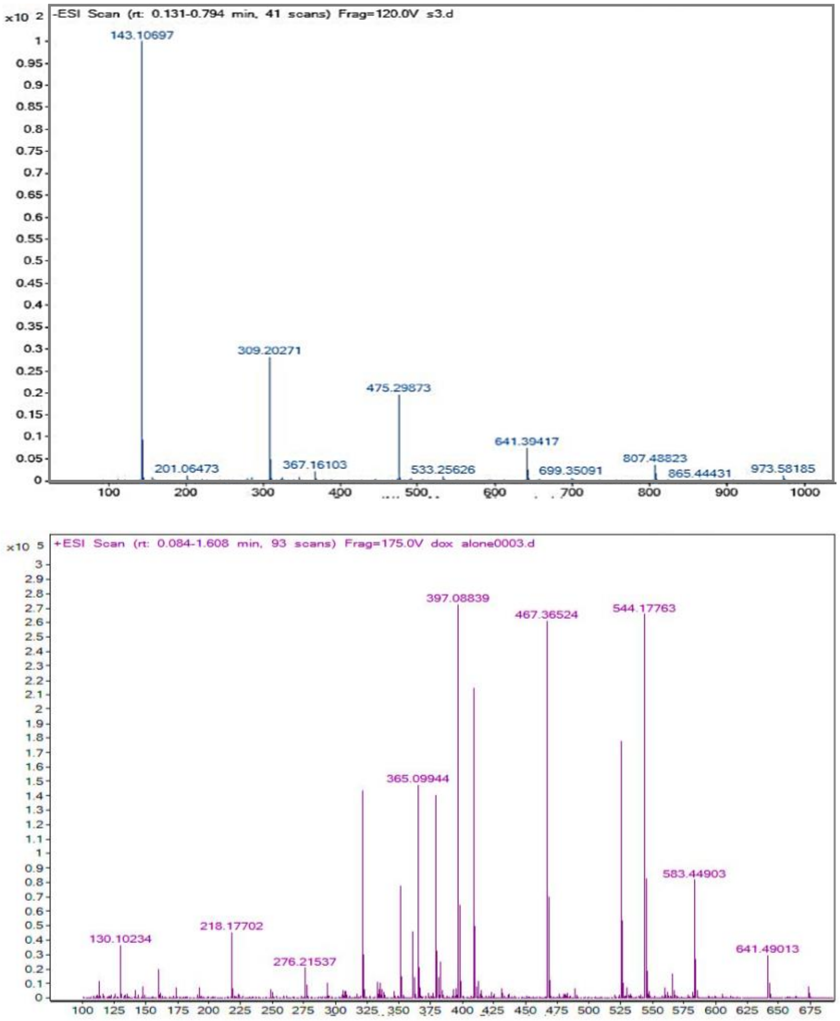

Figure S5. QTOF data of the drugs used for protein-drug nanoconjugates formation. (A) VPA alone showing molecular ion (M^+) peak at 143nm and (B) Dox alone showing molecular ion (M^+) peak at 397nm and 544nm.

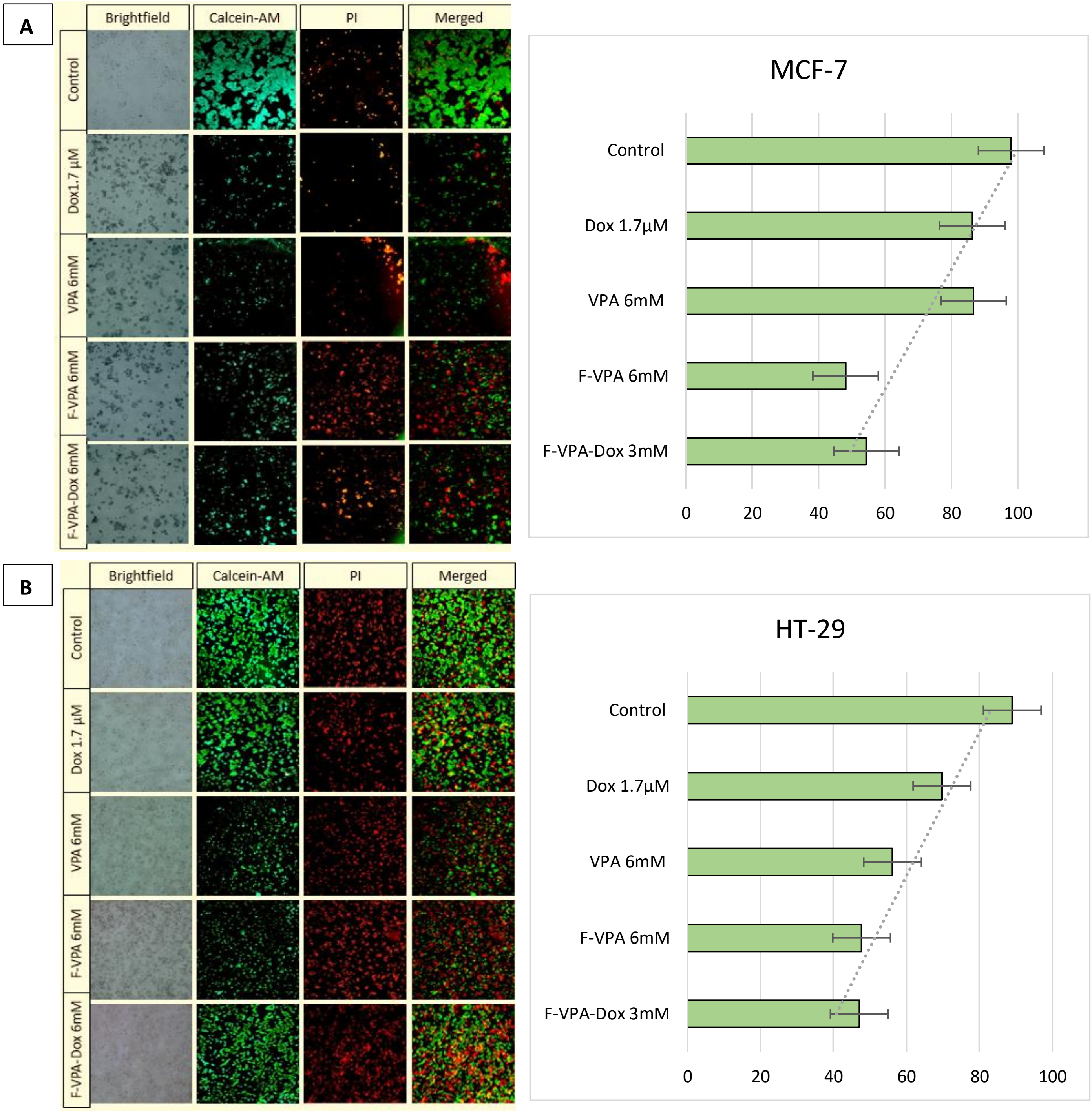

Figure S6. Detailed 2D-monolayer images of cancer cell lines (A) MCF-7 cells and (B) HT-29 cells before and after the treatment of drug alone and protein-drug nanoconjugates.

Table S1. The combination index (CI) was calculated at the indicated concentration of valproic acid (VPA) and doxorubicin (Dox) by using the equation CI = AB / (A × B). Here, AB is the ratio of the combination treatment group to that of the control group; A or B is the ratio of the single drug group to that of the control group. Hence, a CDI value <1 indicates synergism; =1 additive; or >1 antagonism. A CDI value <0.7 indicates significant synergism [70].

Cell Line	Doxorubicin (%)	Valproic Acid (%)	VPA-Dox (mM %)	Combination Index (CI)
MCF-7	60%	58%	70%	0.02
C4-2	65%	72%	75%	0.01
HT-29	10%	30%	90%	0.31

Video S1. Monitoring the spheroid growth for C4-2 cells on different days.

Video S2. Monitoring the spheroid growth for HT29 cells on different days.

Video S3. Monitoring the spheroid growth for MCF7 cells on different days.
